# Model‐based analysis of the influence of catchment properties on hydrologic partitioning across five mountain headwater subcatchments

**DOI:** 10.1002/2014WR016147

**Published:** 2015-06-09

**Authors:** Christa Kelleher, Thorsten Wagener, Brian McGlynn

**Affiliations:** ^1^ Department of Earth and Ocean Sciences, Nicholas School of the EnvironmentDuke UniversityDurhamNorth CarolinaUSA; ^2^ Department of Civil EngineeringUniversity of BristolBristolUK; ^3^ Cabot InstituteUniversity of BristolBristolUK

**Keywords:** catchment modeling, comparative hydrology, sensitivity analysis, DHSVM

## Abstract

Ungauged headwater basins are an abundant part of the river network, but dominant influences on headwater hydrologic response remain difficult to predict. To address this gap, we investigated the ability of a physically based watershed model (the Distributed Hydrology‐Soil‐Vegetation Model) to represent controls on metrics of hydrologic partitioning across five adjacent headwater subcatchments. The five study subcatchments, located in Tenderfoot Creek Experimental Forest in central Montana, have similar climate but variable topography and vegetation distribution. This facilitated a comparative hydrology approach to interpret how parameters that influence partitioning, detected via global sensitivity analysis, differ across catchments. Model parameters were constrained a priori using existing regional information and expert knowledge. Influential parameters were compared to perceptions of catchment functioning and its variability across subcatchments. Despite between‐catchment differences in topography and vegetation, hydrologic partitioning across all metrics and all subcatchments was sensitive to a similar subset of snow, vegetation, and soil parameters. Results also highlighted one subcatchment with low certainty in parameter sensitivity, indicating that the model poorly represented some complexities in this subcatchment likely because an important process is missing or poorly characterized in the mechanistic model. For use in other basins, this method can assess parameter sensitivities as a function of the specific ungauged system to which it is applied. Overall, this approach can be employed to identify dominant modeled controls on catchment response and their agreement with system understanding.

## Key Points

- Sensitivity analysis of an uncalibrated model constrained by expert knowledge
- Sensitive parameters are similar across subcatchments but vary seasonally
- A subset of snow, soil, and vegetation parameters control partitioning

## 1 Introduction

Headwater streams are the smallest streams within a stream network. Despite their size, headwaters are responsible for the ecological integrity of surrounding and downstream waters via the ecosystem services that they provide [*Leopold et al*., 1964; *Lowe and Likens*, 2005]. This is in part due to their abundance, as Strahler first and second order streams represent upward of 70% of total stream length across the United States river network [*Nadeau and Rains*, 2007]. They also represent a significant linkage between the terrestrial system and the downstream environment, exporting water, organic matter, and nutrients to the rest of the stream network [*Wipfli et al*., 2007]. Headwaters exhibit high biodiversity, provide areas of habitat for sensitive species, and serve as havens from downstream areas during water and temperature‐stressed periods [*Gomi et al*., 2002; *Meyer et al*., 2007; *Kelleher et al*., 2011].

While numerous studies have acknowledged the hydrological and ecological importance of headwater streams [*Nadeau and Rains*, 2007; *Freeman et al*., 2007; *Lowe and Likens*, 2005], the connections among physical setting, hydrological behavior, and ecological integrity within them are not well understood [*Gomi et al*., 2002; *Wagener et al*., 2008]. Headwater streams are the least instrumented portion of the stream network and are even sometimes unmapped because of their small size [*Poff et al*., 2006; *Freeman et al*., 2007]. As such, historical information about headwater streams is lacking and limited to a few well‐instrumented locations across the United States [*Jones et al*., 2012]. Studies at these experimental catchments do provide important information about headwater function but are typically site specific [e.g., *Swank and Miner*, 1968; *Elliott and Vose*, 2011]. What makes these subcatchments particularly relevant to studying headwater hydrology is that they are often divided into multiple monitored subcatchments, offering the opportunity to study how physical differences influence behavior under identical climate [e.g., *Jones and Post*, 2004; *Jencso and McGlynn*, 2011].

Comparative hydrology [*Falkenmark and Chapman*, 1989], which has been recently advocated as one future path for hydrological research [*Wagener et al*., 2007; *Sivapalan*, 2009; *Wagener et al*., 2010; *Blöschl et al*., 2013], is based on the concept that the hydrologic behavior of a given catchment reflects the evolution and current state of climate, geology, vegetation, and terrain [*Falkenmark and Chapman*, 1989]. Thus, a comparative hydrology approach connects variability in physical setting to differences in hydrological behavior. Other comparative studies have highlighted the relationships between physical setting and hydrologic behavior for specific types of catchment response (e.g., evapotranspiration) [*Thompson et al*., 2011] and for different temporal scales (e.g., event, seasonal, and interannual streamflow) [*Jefferson et al*., 2008]. These studies have either been model driven [*Tague and Grant*, 2009; *Christensen et al*., 2008; *Carrillo et al*., 2011] or empirical [*Jencso and McGlynn*, 2011; *Sawicz et al*., 2011], with some using a combination of the two [*Nippgen et al*., 2011; *Tague et al*., 2008]. The contribution of these studies is their attempt to generalize relationships in terms of dominant processes, which makes their conclusions transferrable to other unmonitored sites.

Our limited ability to transfer information reliably to ungauged catchments is still a major bottleneck to understanding headwater behavior and to hydrology in general [*Jones and Swanson*, 2001; *Blöschl et al*., 2013]. To address this need, we developed and tested a comparative hydrology framework for identifying dominant controls in headwater systems. We use an uncalibrated, physically based hydrologic model as a tool for identifying primary influences (model parameters) on hydrologic response (model predictions). We assume that the mechanistic model is (in principle) a realistic representation of the dominant processes occurring in the catchment, and that a priori distributions of the physically based parameters can be defined from our knowledge of the catchment physical characteristics alone [*Carrillo et al*., 2011]. Within this framework, we used global sensitivity analysis to link variability in model output to parameters that describe soils, vegetation, and snow properties of a given catchment. Sensitive parameters represent dominant controls on system behavior and will vary with static model inputs, including the distribution of topography and vegetation.

The scope of our study was to apply this framework to interpret similarities and differences in dominant controls on hydrologic partitioning across five adjacently located headwater subcatchments within the Tenderfoot Creek Experimental Forest in central Montana. Hydrologic partitioning refers to the partitioning of water across the catchment environment; here we use it to refer to the separation of precipitation into different catchment storages and subsequent fluxes from these storages. We investigated parameter controls on multiple catchment model states, including fluxes (streamflow and evapotranspiration) and storages (soil moisture and snow water equivalent) for seasonally varying conditions. Catchment processes were simulated with the Distributed Hydrology‐Soil‐Vegetation Model (DHSVM), a physically based, distributed model that has been extensively applied to mountainous western headwater catchments and is ideally suited for this application [e.g., *Doten et al*., 2006; *Jost et al*., 2009; *Du et al*., 2013; and many more]. While this was a purely model‐based evaluation, the interpretation of model controls was performed with an understanding of subcatchment functioning from extensive fieldwork, monitoring, and empirical analyses that have been performed in Tenderfoot Creek and summarized in numerous publications [*Jencso et al*., 2009, 2010; *Payn et al*., 2009, 2012; *Pacific et al*., 2010; *Emanuel et al*., 2010; *Jencso and McGlynn*, 2011; *Nippgen et al*., 2011]. We leveraged these extensive measurements to evaluate whether this framework can effectively identify dominant controls, as compared to our perception of important catchment processes and the parts of the landscape that define them. Instead of using these observations to evaluate how well the model represents the catchment via error metrics alone, we emphasize the level of information one can obtain in an ungauged catchment. In the absence of observations, this type of approach has the potential to identify important catchment characteristics needed to predict hydrologic behavior as well as catchments where the model is a poor representation of the system.

## 2 Study Area and Data

### 2.1 Description of the Tenderfoot Creek Experimental Forest

**Figure 1:**
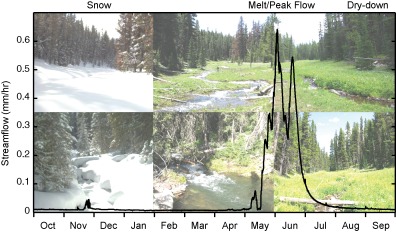
Stringer Creek streamflow plotted through time for the 2008 water year, with pictures corresponding to different periods of hydrological behavior. Photo credits to F. Nippgen and C. Kelleher.

Tenderfoot Creek Experimental Forest is located in central Montana (Figure 1). The outlet of Tenderfoot Creek, a tributary to the Smith River, drains five adjacent headwater catchments through first order streams. Tenderfoot Creek has a continental climate, with some variability in weather with elevation. Precipitation is an average 880 mm yr^−1^ but ranges from 594 at low elevations to 1050 at high elevations [*Jencso and McGlynn*, 2011]. Approximately 75% of precipitation falls as snow, with melt occurring in May or June. Runoff is highest at snowmelt and declines through the summer and into the winter months. Peak soil moisture coincides with peak runoff and declines throughout the growing season [*Riveros‐Iregui et al*., 2007; *Emanuel et al*., 2010] (Figure 1).

**Figure 2:**
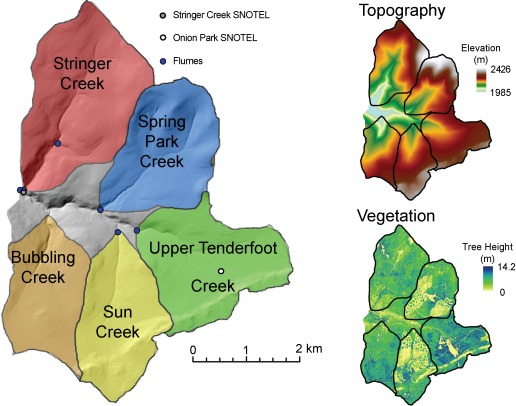
Tenderfoot Creek catchment and subcatchments, (1) Stringer Creek, (2) Spring Park Creek, (3) Upper Tenderfoot Creek, (4) Sun Creek, and (5) Bubbling Creek, and locations of SNOTEL sites and flux towers. Elevation and tree height are also shown across the subcatchments.

**Figure 3:**
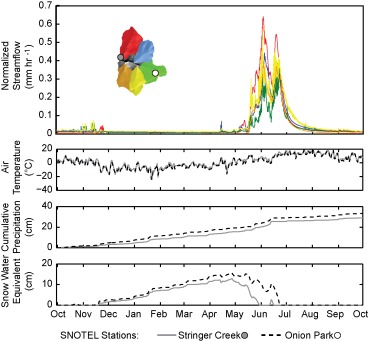
Meteorological and streamflow data for the 2008 water year.

Tenderfoot Creek spans an area of 23 km^2^, with subcatchments ranging from 3.18 km^2^ to 5.54 km^2^ (Figure 2). Subcatchments include Bubbling Creek (BUB), Sun Creek (SUN), Upper Tenderfoot Creek (UTC), Spring Park Creek (SPC), and Stringer Creek (STR). Flumes are located at Tenderfoot Creek and each of the five additional subcatchments via Parshall or H‐Flumes at both hourly and 15 minute resolution. Climate measurements, including air temperature, relative humidity, wind speed, and precipitation, are recorded at two SNOTEL sites dating back to 1991, as well as four H_2_O/CO_2_ eddy‐covariance towers with full energy budget instrumentation, which have been operational since 2005 [*Jencso et al*., 2009; *Emanuel et al*., 2010; *Jencso and McGlynn*, 2011] (Figure 2). Streamflow, normalized to catchment area, for the five subcatchments and climate data from two snow telemetry (SNOTEL) sites located in Lower Stringer and Upper Tenderfoot Creek are shown in Figure 3 for the 2008 water year.

Geology varies across the catchment, with Wolsey shale and Flathead sandstone dominating higher elevations and granite gneiss at lower elevations. Soil depth and type varies across riparian and hillslope settings [*Holdorf*, 1981; *Jencso et al*., 2009]. Across the hillslopes, soils are loamy skeletal, mixed typic Cryochrepts. Riparian soils are characterized as clayey, mixed Aquic Cryboralfs. Average soil depths are approximately 1 m, as estimated during installation of about 160 wells and piezometers [*Jencso et al*., 2009]. Lodgepole pine overstory and grouse whortleberry understory dominate hillslopes, and bluejoint reedgrass is predominant in the riparian zone [*Farnes et al*., 1995; *Mincemoyer and Birdsall*, 2006]. Further descriptions of the Tenderfoot Creek Experimental Forest can be found in *Farnes et al*. [1995; climate and vegetation], *Reynolds* [1995; geology], and *Mincemoyer and Birdsall* [2006; vegetation].

Past work in Tenderfoot Creek has utilized variability in landscape structure and setting to understand biogeochemical cycling [*Pacific et al*., 2010], hydrological connectivity [e.g., *Jencso and McGlynn*, 2011], and ecohydrological catchment response through time [*Emanuel et al*., 2010; *Kaiser et al*., 2013]. More recently, hydrological studies and modeling have focused on the influence of variable catchment structure on runoff generation [*Jencso et al*., 2009, 2010; *Jencso and McGlynn*, 2011] and controls on response times across subcatchments [*Nippgen et al*., 2011]. Work by *Jencso and McGlynn* [2011], *Nippgen et al*. [2011], and *Payn et al*. [2012] has found that similarities and differences in hydrologic behavior across subcatchments can be explained partially by geology, vegetation, and catchment structure. While some catchments have been found to behave somewhat similarly (Upper Tenderfoot Creek, Middle Stringer Creek, Lower Tenderfoot Creek, Bubbling Creek, and Lower Stringer Creek), others have been identified as relative outliers (Sun Creek, Spring Park Creek) [*Nippgen et al*., 2011]. Across these studies, the characteristics of these subcatchments that most influenced different types of hydrologic behavior varied with temporal scale. Most previous work at this site has separated hydrologic behavior into three distinct periods (Figure 1): snowmelt [e.g., *Pacific et al*., 2010], a summer recession, and low or no flow periods coinciding with the fall and winter when snow accumulates [*Jencso and McGlynn*, 2011].

## 3 The Distributed Hydrology‐Soil‐Vegetation Model (DHSVM)

### 3.1 Description of DHSVM

DHSVM is a physical, grid‐based hydrologic model that computes the water and energy balance for each cell within a spatially discretized catchment. Spatially distributed parameters represent topography, soil depth, and soil and vegetation type. Soil and vegetation properties, used to represent different land covers, are distributed by class types assigned to each cell. Meteorological inputs to the model include precipitation, incoming shortwave radiation, incoming longwave radiation, relative humidity, wind speed, and air temperature. The original version of the model is described in *Wigmosta et al*. [1994], but a more comprehensive description of recent updates in model routines can be found in *Wigmosta et al*. [2002].

### 3.2 DHSVM Components

DHSVM includes seven different modules used to represent the flow of water for a given catchment: evapotranspiration, snowpack accumulation and melt, canopy snow interception and release, unsaturated moisture movement, saturated subsurface flow, surface overland flow, and channel flow [*Wigmosta et al*., 1994, 2002]. The one‐dimensional vertical water balance solved for each cell includes the effects of precipitation, interception, evaporation, transpiration, infiltration, and soil moisture storage. Evapotranspiration is computed via a Penman‐Monteith approach, with vegetation represented with either an understory (undergrowth] or overstory (canopy) and understory [*Wigmosta et al*., 2002]. Each cell contains multiple soil layers with fractions of roots from the overstory and/or understory. Transpiration is a function of soil moisture and the root zone fraction per soil layer. Water reaches the ground surface via throughfall, snowmelt, or surface runoff and is infiltrated up to a maximum rate per time step. Once infiltrated, unsaturated moisture movement is simulated laterally with hydraulic conductivity approximated via the Brooks‐Corey relationship and vertically via percolation according to Darcy's Law [*Wigmosta et al*., 1994]. Saturated subsurface flow is routed between cells based on kinematic or diffusion approximations [*Wigmosta et al*., 1994; *Wigmosta and Lettenmaier*, 1999]. The water table depth for each cell is computed based on percolation and saturated and unsaturated water redistribution from neighboring cells. Streamflow is routed using a spatially delineated stream network with channel routing via a cascade of linear reservoirs [*Wigmosta et al*., 1994; *Wigmosta and Lettenmaier*, 1999]. The model also includes a two‐layer snow accumulation and melt model, described in *Storck and Lettenmaier* [1999] and *Storck* [2000]. The two‐layer energy and mass balance additionally includes a canopy snow interception and release model, which incorporates the effects of snow interception, meltwater drip, and mass release from the canopy. Precipitation is partitioned into rain, snow, or a mixture of the two based on two temperature thresholds, a minimum temperature at which rain can occur and a maximum temperature at which snow can occur.

### 3.3 DHSVM Application to Tenderfoot Creek Experimental Forest

A grid resolution of 10 m was used to define all spatial data sets, as previous studies have found this to be an appropriate resolution for observing and characterizing hydrologic and biochemical processes [*Jencso et al*., 2009; *Pacific et al*., 2010]. Elevation grids for each subcatchment were generated by resampling the Airbourne Laser Swath Mapping (ALSM) data to a 10 m digital elevation model (DEM). Topography represents a source of predefined variability between the subcatchments. It is not varied within the sensitivity analysis, like the soil and vegetation parameters, but does influence how water is partitioned across the landscape. Topographic, vegetative, and stream network characteristics for each of the subcatchments are summarized in Table 1.

**Table 1:** Physical Characteristics for the Tenderfoot Creek Experimental Basins

		Elevation (m)			Vegetation (%)				
Subcatchment	Drainage Area (km^2^)	Min	Max	Mean	<2 m	2–10 m	10+	Stream Length (m)	Drainage Density (km^−1^)
Stringer Creek	5.5	1996	2426	2217	22.8	77.2	0.009	4596	0.812
Spring Park Creek	4.5	2103	2426	2250	37.3	62.7	0.015	3609	0.784
Upper Tenderfoot Creek	4	2153	2358	2262	17.1	82.8	0.128	2472	0.641
Sun Creek	3.2	2135	2365	2262	44.2	55.8	0.022	1920	0.581
Bubbling Creek	3.6	2040	2361	2212	10.0	90.0	0.038	2574	0.705

Our understanding of the catchment suggests that vegetation height influences catchment functional responses to snow, wind resistance, and evapotranspiration [*Keane et al*., 2005; *Jensen et al*., 2008] and organizes hydrologic response across the catchments [*Nippgen et al*., 2011; *Jencso and McGlynn*, 2011]. Therefore, height was the primary factor used to delineate vegetation classes that distribute vegetation parameters across the basins. Vegetation heights were delineated across the catchments as the difference between first returns and ground elevations in 1 m resolution ALSM data. Canopy and snow return differences at 1 m resolution were averaged to 10 m and then grouped into three different vegetation classes based on height. Cells with vegetation at heights less than 2 m were designated as having just an understory consisting of grasses and shrubs. Cells with vegetation heights greater than 2 m were assumed to have both an overstory, consisting of Lodgepole pine forest, and an understory. The Lodgepole pine forest was divided into cells with medium‐sized trees, with heights of less than 10 m, and tall trees, with heights greater than 10 m. The distribution of trees versus grasses and shrubs varies across the subcatchments due to past clear cutting in Spring Park and Sun Creek, as can be seen in Figure 2. Clear‐cut subcatchments exhibit about 37% (Spring Park) and 44% (Sun) of vegetation at heights less than 2 m (Table 1).

While exact species of vegetation types are known from Tenderfoot field surveys and previous publications, our perception of this distribution is that there is limited species variability and functional variability across the entirety of the catchment [*Ahl et al*., 2008]. As such, we did not further subdivide vegetation classes based on species. However, we do assume that the ground cover will functionally behave differently than canopy trees, so parameter values were additionally varied between these two groups as described above. Ranges for vegetation parameters were taken from a number of different sources and are defined in Table 2. When possible, these parameters were constrained using the actual vegetation species described in Tenderfoot Creek and noted in section 2.1. Vegetation heights for the grass and shrub cells were varied between 0 and 2 m. ALSM data were additionally used to constrain vegetation height and the fractional canopy coverage for cells classified as having tree cover. Vegetation heights were calculated using the mean plus or minus one standard deviation for within both vegetation classes (2–10 m and 10+ m) across each catchment. As these values hardly varied across the subcatchments, the overall minimum and maximum values across all five catchments were then applied to represent the range of local variability of these values across the entirety of Tenderfoot Creek. The same process was applied for the parameter representing the fraction of canopy covering each cell, calculated for each 10 m cell as the fraction of 1 m cells with a canopy (height > 2 m).

**Table 2:** Metrics Selected to Assess Model‐Predicted Output^a^

	Metric Name		Units	Calculation	Importance to Behavior/Function
Streamflow	Runoff ratio			Total annual streamflow divided by precipitation	Annual measure of the water balance
	Maximum streamflow		m^3^/s	Annual maximum	Magnitude of highest flow is important to watershed response
	Timing of Q_MAX_			Julian date of maximum Q	Timing of highest flow is an ecological cue
	Base flow index			The annual average of the 7 day low flows	Magnitude of base flow, which assesses low flows before and after snowmelt
	Slope of the flow duration curve		m^3^/s	The slope of the line between the twentieth percentile and sixtieth percentile flow	Assesses basin flashiness via the distribution and transition between high and low flow values
	Coefficient of variation			The standard deviation divided by the mean streamflow	Measures variability in the streamflow record
Evapotranspiration (ET)	Summed annual evapotranspiration		m	Summed evapotranspiration from 1 Oct through 30 Sep	Total ET informs ecosystem productivity
	Summed growing season evapotranspiration		m	Summed evapotranspiration from 1 Jun through 1 Aug	Growing season ET measures behavior across the period of highest productivity
Soil moisture	Top layer	Average	%	Annual average	Shallow soil response during wet (maximum), dry (minimum), and average conditions
		Maximum	%	Annual maximum	
		Minimum	%	Annual minimum	
	Middle layer	Average	%	Annual average	Saturated/unsaturated transitional soil response to wet (maximum), dry (minimum), and average conditions
		Maximum	%	Annual maximum	
		Minimum	%	Annual minimum	
	Bottom layer	Average	%	Annual average	Deep soil/saturated system response during wet (maximum), dry (minimum), and average conditions
		Maximum	%	Annual maximum	
		Minimum	%	Annual minimum	
Snow water equivalent (SWE)	Peak SWE		m	Maximum value of SWE across the year	Measures the total annual SWE in terms of its peak value
	Timing of peak			Julian date of maximum SWE	Informs the beginning of the melt period
	SWE storage		m × h	Area under the SWE curve	Represents total annual SWE storage
	Maximum 3 day melt		m/h	The maximum melt, computed over a 3 day period	Captures the greatest melt rate and therefore input to the ground

Q: streamflow (m^3^/s); θ_1_: soil moisture, top soil layer (%); θ_2_: soil moisture, middle soil layer (%); θ_3_: soil moisture, bottom soil layer (%); ET: evapotranspiration (mm); SWE: snow water equivalent (m).

Model representation of soil functionality is based on the field observation‐informed perception that there is modest variability in soil type and depth in space. What variability there is has not yet been mapped across the Tenderfoot Creek catchment. Soil type and depth was informed instead using the CONUS‐SOIL data set [*Miller and White*, 1998]. CONUS soil data are at a much coarser resolution than were used to model Tenderfoot Creek. Therefore, single type with a uniform soil depth was applied across all subcatchments, representative of soil type MT064 in the CONUS system. Soil properties for this soil type were constrained based on CONUS‐reported properties, model‐defined default values, and from the Soil Water Characteristics package [*Saxton and Rawls*, 2006], which calculates properties based on soil texture in terms of sand, silt, and clay fractions reported by CONUS. For this application, any variability in soil properties with depth was small, and soil properties were assumed to be constant with depth. Soil parameter ranges and sources are specified in Table 2. Snow parameters include but are not limited to the snow water holding capacity, a general rain and snow leaf area index value, and threshold temperatures that define the upper limit at which snow can occur and the lower limit at which rain can occur.

Meteorological data were prepared at a three‐hourly time step. A time step of 3 hours was selected because it appropriately captured the diurnal fluctuations in weather that results in snowmelt and accumulation without compromising computational efficiency. Missing data were linearly interpolated either using values from the previous and next time step or when long periods were missing, using values from the previous/next day at the same hour. Solar radiation data were obtained from an eddy flux tower located in Stringer Creek (Figure 2) and used as the solar radiation input at both SNOTEL sites. Eddy flux tower solar radiation was scaled to SNOTEL locations based on landscape position. Solar radiation is spatially distributed within DHSVM in terms of an annual topographic shading map as well as through monthly averaged maps at three‐hourly time steps that account for slope and aspect. In this application, the combination of extremely low winter air temperatures and monthly averaged shading maps produced issues with model‐predicted surface temperatures. To address these issues, we used the fully distributed monthly shading routines for the majority of the year, and three‐hourly averaged shading maps for the winter months (November–January for Stringer Creek and Bubbling Creek; October–January for Spring Park Creek, Upper Tenderfoot Creek, and Sun Creek). Thus, we still account for variability in solar radiation over large portions of the year when it most affects melt processes (April–June) and summer drydown (July–September).

Longwave radiation data were estimated for both SNOTEL locations based on air temperature and relative humidity following *Dingman* [2002]. Meteorological observations were distributed across the subcatchments using an inverse distance approach contained within DHSVM. The Stringer Creek SNOTEL station, located within the Stringer Creek subcatchment, is at an elevation of 1996 m, while the Onion Park SNOTEL station, located in the Upper Tenderfoot Creek subcatchment, is at an elevation of 2259 m. These sites span a significant elevation gradient within Tenderfoot Creek and likely capture any variations in weather with elevation. To include the variability of air temperature with elevation, we calculate and force the model with an air temperature lapse rate between the two SNOTEL stations.

## 4 Sensitivity Analysis

Sensitivity analysis can be a useful tool for understanding relationships between model inputs and outputs [*Saltelli et al*., 2008]. In catchment modeling applications, this translates to understanding how uncertainty in catchment properties parameterized in the model (e.g., soil porosity and rooting depth) influence model‐derived catchment behavior (e.g., streamflow) [*van Werkhoven et al*., 2008]. The most thorough strategy for such a study is global, variance‐based sensitivity analysis [*Tang et al*., 2007], but it requires a large number of model runs if the model has many parameters. For physically based, distributed models, which often include many parameters and have longer run‐times than lumped models, global, variance‐based sensitivity analysis may not be feasible. Therefore, we use the Method of Morris, a global sensitivity analysis that is often applied as a screening method to identify important parameters with respect to a given output metric [*Morris*, 1991].

### 4.1 Method of Morris

The Method of Morris is a global sensitivity analysis approach that is used to identify or “screen” factors that are nonnegligible to predicting model output [*Morris*, 1991]. In this sense, as it is more concerned with screening important parameters than quantifying sensitivity in terms of variance, it requires far fewer model runs than variance‐based sensitivity analyses [*Campolongo et al*., 2007; *Herman et al*., 2013]. It is often used in conjunction with a variance‐based sensitivity analysis, with the Morris Method first applied to reduce the size of the parameter space [*Saltelli et al*., 2004]. Comparisons with other sensitivity analyses indicate that the Method of Morris satisfactorily assesses the importance of different model factors [*Campolongo and Saltelli*, 1997].

The method itself consists of multiple, random one‐factor‐at‐a‐time (OAT) experiments, also termed “elementary effects,” that are repeated across many paths through the parameter space in order to account for individual parameter influence and interactions [*Saltelli et al*., 2005]. For a single parameter *x_i_*, an elementary effect *EE_i_* is calculated as the change in parameter value with model output along a trajectory with a grid of size Δ*_i_*. Model output is approximated at *p* grid points (for a model with *p* parameters) between a parameter minimum and maximum. A single elementary effect is calculated as

(1) $$EE_{i}=\frac{f\left(x_{1},\ldots ,x_{i}+\Delta_{i}\ldots ,x_{p}\right)-f(x)}{\Delta_{i}}$$

where *f*(*x*) is the previous trajectory point. Morris statistics are based on many such elementary effects approximated for multiple trajectories through the parameter space. Two different sensitivity indices are computed per parameter from these many localized measures of sensitivity. The first is the mean of the Elementary Effects *μ*, which assesses the importance of a given parameter to predicting a given model output metric. The second is the standard deviation of the Elementary Effects *σ*, which assesses the level of interaction between a given model factor and other model factors in predicting a given model output metric. Both *μ* and *σ* are computed for all model parameters for a given model output. It is worth noting that the importance of a given model input factor should be assessed using both *μ* and *σ*, as the value of *μ* alone may underestimate the importance a given model factor in the case that it is nonmonotonic [*Campolongo et al*., 2007]. Higher values of both metrics for a given model factor indicate higher importance and higher degree of interactions, respectively. In addition to calculation of the sensitivity metrics, confidence intervals were also calculated via bootstrapping for each sensitivity measure, per parameter, per metric, to consider uncertainty in these estimates.

For this application, we used the Method of Morris within the Sensitivity Analysis for Everyone (SAFE) Toolbox [*Pianosi et al*., 2015] to investigate 54 model factors for ranges specified in Table 2. The parameter space was sampled using the Sobol' sampling strategy, an improvement over the original randomized sampling strategy of the Method of Morris as presented in *Morris* [1991] and *Campolongo et al*. [2007]. The total number of model evaluations *N* performed is equal to

(2) N=r⋅(p+1)

where *p* is equal to the number of model factors being investigated and *r* is the number of sampling points. Literature recommendations for *r* vary between 10 and 50 [*Campolongo et al*., 2007; *Herman et al*., 2013], but increasing this value will only provide more reliability. We applied the Method of Morris to a number of different response metrics (described in section 4.2) that capture streamflow, snow water equivalent, soil moisture, and evapotranspiration behavior during different periods of the year. Robustness was assessed via confidence intervals computed using 1000 resamples for bootstrapping. Sensitivity metrics were compared across subcatchments and across response metrics.

### 4.2 Model Output Metrics

We use the Method of Morris to assess the impact of model parameters on a range of catchment behaviors (Table 3). It is important to note that all metrics were calculated solely based on three‐hourly model‐predicted output, i.e., not by comparing to an observed system response such as streamflow. Output was obtained either as an average across the subcatchments (evapotranspiration, soil moisture, and snow water equivalent) or as an output at the subcatchment outlet (streamflow). Metrics were selected to assess model behavior to capture time‐varying responses and partitioning of water across the subcatchments, and to capture behavior that is hydrologically important to this area of Montana. Hydrologic response in Tenderfoot Creek occurs during three “hydrologic seasons”—a period of snow accumulation (October–April), a period during which snowmelt drives high streamflow (May), and a summer dry‐down corresponding to the growing season (June–July). Snowmelt provides the major water input that drives streamflow and supplies storage to the catchment. Following melt and snowmelt driven runoff, significant dry‐down across the catchment is mediated by evaporation and transpiration. Responses and partitioning vary across different seasons; metrics were selected to capture this seasonality and how it may vary across the different subcatchments.

**Table 3:** Parameter Ranges and References for Ranges for Sensitivity Analysis^a^

Type	#	Parameter	Source/Reference	Minimum	Maximum
Snow/climate	1	Snow water capacity	*Kattelmann et al*. [1991]	0.01	0.08
	2	Rain LAI multiplier	*Czarnowski and Olszewski* [1968]	0.0002	0.001
	3	Snow LAI multiplier	*Storck* [2000]	0.0002	0.0025
	4	Minimum intercepted snow	Model default ± 1 order of magnitude	0.001	0.01
	5	Snow threshold	*Dai* [2008]	0	2
	6	Rain threshold	*Dai* [2008]	−2	0
Soil	7	Lateral conductivity	Limited information—kept wide	1 × 10^−4^	1 × 10^−2^
	8	Exponential decrease		0.5	5
	9	Maximum infiltration	*Akan* [1993]	3.6 × 10^−5^	5.38 × 10^−4^
	10	Capillary drive	*Morel‐Seytoux and Nimmo* [1999]	0.03	0.6
	11	Surface albedo	*Dingman* [2002]	0.2	0.3
	12	Porosity	CONUS, *Miller and White* [1998]	0.38	0.47
	13	Pore size distribution	*Rawls et al*. [1982]	0.07	0.559
	14	Bubbling pressure	*Rawls et al*. [1982]	0	1.24
	15	Field capacity	*Saxton and Rawls* [2006]	0.15	0.25
	16	Wilting point	*Saxton and Rawls* [2006]	0.07	0.15
	17	Bulk density	*Saxton and Rawls* [2006]	1390	1650
	18	Vertical conductivity	*Meyer et al*. [1997]	1.42 × 10^−10^	2.02 × 10^−4^
	19	Thermal conductivity	*Abu‐Hamdeh and Reeder* [2000], *Ochsner et al*. [2001]	0.3	0.8
	20	Thermal capacity	*Ochsner et al*. [2001]	1 × 10^6^	3 × 10^6^
	21	Mannings n	*Sturm* [2010]	0.11	0.35
Undergrowth	22	Maximum snow interception capacity	*Breuer et al*. [2003], *Cuo et al*. [2011]	0.05	0.2
	23	Mass release drip ratio	*Wigmosta et al*. [1994], model default	0	1
	24	Snow interception efficiency	*Cuo et al*. [2011], model default	0	1
	25	Height	Basin ALSM data	0	1.2
	26	Maximum resistance	*Cuo et al*. [2011], *Land Data Assimilation Systems* (*LDAS*) [[Link]]	500	1000
	27	Minimum resistance	*Rosenzweig and Abramopoulos* [1997], *Zhou* [2011], *LDAS* [[Link]]	100	175
	28	Moisture threshold	*Meyer et al*. [1997], *Cuo et al*. [2011]	0.115	0.165
	29	Vapor pressure deficit	*Fetcher* [1976], *Wigmosta et al*. [1994]	200	4000
	30	Rpc	*Chen* [1996]	0.1	1
	31	Root fraction, layer 1	*Zeng* [2001]	0.25	0.45
	32	Root fraction, layer 2	*Zeng* [2001]	0.45	0.65
	33	Monthly LAI	*LDAS* [[Link]], *Mitchell et al*. [2004]	0.65	1.35
	34	Monthly albedo	*LDAS* [[Link]], *Mitchell et al*. [2004]	0.1	0.23
Lodgepole pine	35	Fractional coverage	Basin ALSM data	0.287	0.575
	36	Trunk space	*Temesgren et al*. [2005]	0.37	0.79
	37	Aerodynamic attenuation	*Cionco* [1972], *Thanapakpawin et al*. [2006], *Cuo et al*. [2011]	0.3	4.25
	38	Radiation attenuation	*Storck* [2000]	0.1	0.2
	39	Maximum snow interception capacity	*Breuer et al*. [2003], *Cuo et al*. [2011]	0.05	0.2
	40	Mass release drip ratio	*Wigmosta et al*. [1994], model default	0	1
	41	Snow interception efficiency	*Cuo et al*. [2011], model default	0	1
	42	Height	Basin ALSM data	2	7.5
	43	Maximum resistance	*Fetcher* [1976], *Cuo et al*. [2011], *LDAS* [[Link]]	500	3000
	44	Minimum resistance	*Rosenzweig and Abramopoulos* [1997], *Zhou* [2011], *LDAS* [[Link]]	150	300
	45	Moisture threshold	*Meyer et al*. [1997], *Cuo et al*. [2011]	0.115	0.165
	46	Vapor pressure deficit	*Fetcher* [1976], *Wigmosta et al*. [1994]	200	4000
	47	Rpc	*Chen* [1996]	0.1	1
	48	Root fraction, layer 1	*Rivard et al*. [1990], *Zeng* [2001]	0.65	0.15
	49	Root fraction, layer 2	*Rivard et al*. [1990], *Zeng* [2001]	0.75	0.25
	50	Monthly LAI	*LDAS* [[Link]], *Mitchell et al*. [2004]	0.5	1.5
	51	Monthly albedo	*LDAS* [[Link]], *Mitchell et al*. [2004]	0.06	0.23
Tall Lodgepole pine	52	Fractional coverage	Basin ALSM data	0.322	0.594
	53	Height	Basin ALSM data	10	14.7

Parameters are grouped by the part of the model they relate to and referenced as such in the text.

Controls on streamflow behavior are assessed using six different metrics, applied over a year of simulation following a model warm‐up period:

(1) The runoff ratio (Q_RR_), equal to the mean annual volume of streamflow divided by the annual volume of precipitation, which assesses the long‐term partitioning of precipitation into streamflow.

(2) The coefficient of variation in streamflow (Q_CV_), calculated as the standard deviation divided by the mean of the instantaneous three‐hourly streamflow time series, which assesses the variability in streamflow across the year.

(3) The base flow index (Q_BFI_), calculated as the mean of all 7 day minimum flows, which quantifies the magnitude of base flow behavior occurring before melt and in late summer after dry‐down.

(4) The slope of the flow duration curve (Q_SFDC_), calculated as the slope between the tenth percentile and fiftieth percentile streamflow values (as defined from the hydrograph), and representative of many of the transitory flow conditions when the catchment “wets up” during melt and “dries down” through the summer period.

(5) The maximum streamflow magnitude (Q_MAX_), obtained as a single maximum value from the time series, capturing the peak value of streamflow which is typically influenced by the magnitude of snow water equivalent.

(6) The timing of peak streamflow (Q_T,MAX_), obtained as the Julian date for the single maximum value extracted from the time series and representative of the timing of melt.

The streamflow metrics selected for analysis include commonly evaluated annual metrics (Q_RR_, Q_CV_) as well as seasonal metrics that capture the functional behavior and seasonal variability in Tenderfoot Creek following periods identified as important by Tenderfoot Creek researchers [*Jencso and McGlynn*, 2011].

Evapotranspiration was assessed as an annual sum as well as a growing season (1 June to 1 August) total. Past work has shown a shift in controls on evapotranspiration during the growing season for Stringer Creek [*Emanuel et al*., 2010]. We assessed both sums as a way to detect how controls change at longer and shorter term scales.

Snow water equivalent (SWE) and the melt it supplies are the primary drivers of streamflow and soil moisture replenishment at Tenderfoot Creek. Four metrics were selected to assess the SWE and melt time series. The magnitude and timing of peak SWE were two metrics chosen to capture the overall maximum SWE, identified as an important control on interannual variability in streamflow [*Nippgen et al*., 2011], as well as the timing of melt initiation. We also assessed controls on the SWE storage over time, calculated as the area under the SWE curve, to help capture early and late season dynamics in addition to peak dynamics, and the 3 day maximum melt, used to assess which factors most influence snowpack dynamics during the warm period following peak SWE.

Soil storage follows the wet and dry periods corresponding to snowmelt and eventual late summer dry‐down. Within DHSVM, the soil column is partitioned into three different layers: a top layer (0–20 cm), a middle layer (20–60 cm), and a deep layer (60–100 cm). To capture the controls on soil moisture, we investigated sensitivity with respect to average, maximum, and minimum soil moisture across these three layers. Assessing the sensitivity during wet (maximum), dry (minimum), and average conditions across these three layers allowed us to investigate the relative differences and shifts in controls between soil and vegetation properties, and in which layers and at which times these shifts occurred.

## 5 Results

### 5.1 Overall Results

The model was run for a warm‐up period from 1 April 2006 to 30 September 2007 with an analysis period of 1 October 2007 through 30 September 2008 for each parameter set. The Method of Morris sensitivity analysis was performed for 21 different metrics assessing model‐predicted streamflow, evapotranspiration, snow water equivalent, and soil moisture. A total of 54 model parameters pertaining to snow/climate inputs, vegetation inputs, and soil inputs were investigated with a parameter sample size of 2200 runs (r = 40). Confidence intervals are calculated for both *μ* and *σ* for each parameter, per metric, and per subcatchment.

### 5.2 Identification of Important Parameters Across Metrics

**Figure 4:**
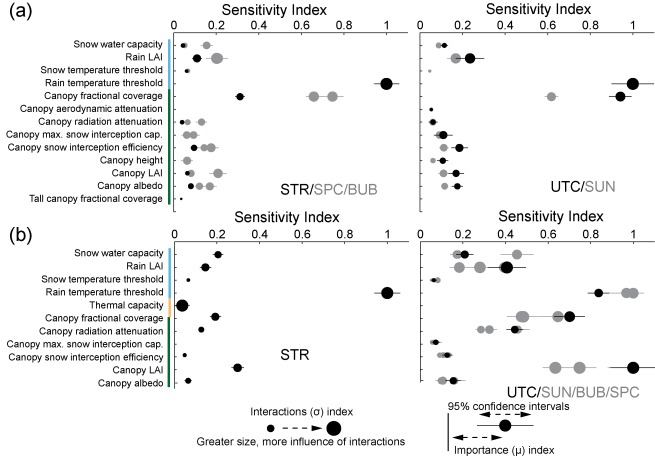
Parameter sensitivities to (a) total annual or (b) growing season evapotranspiration. Abbreviations for subcatchments are STR, Stringer Creek; SPC, Spring Park Creek; UTC, Upper Tenderfoot Creek; SUN, Sun Creek; and BUB, Bubbling Creek. The colors of the abbreviations (black/grey) correspond to the results for each subcatchment.

**Figure 5:**
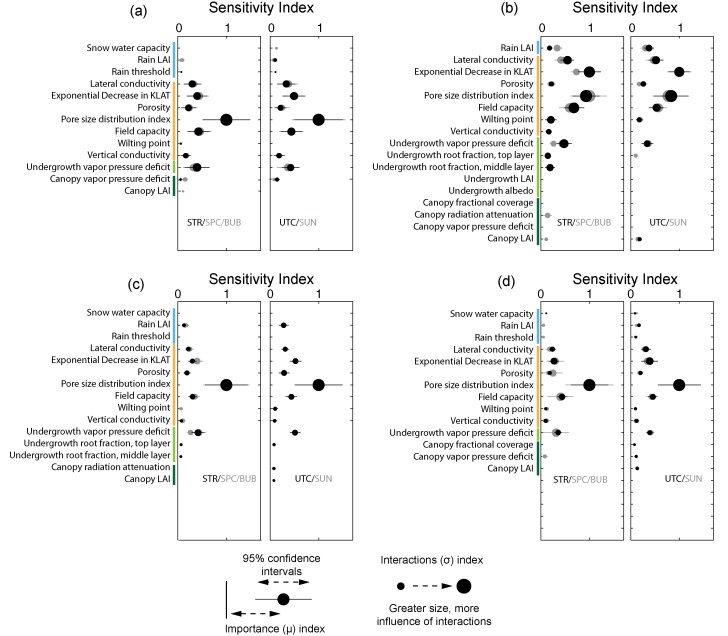
Parameter sensitivities to (a) runoff ratio, (b) slope of the flow duration curve, (c) base flow index, and (d) coefficient of variation for streamflow across the subcatchments. Abbreviations for subcatchments are STR, Stringer Creek; SPC, Spring Park Creek; UTC, Upper Tenderfoot Creek; SUN, Sun Creek; and BUB, Bubbling Creek. The colors of the abbreviations correspond to the results for each subcatchment.

**Figure 6:**
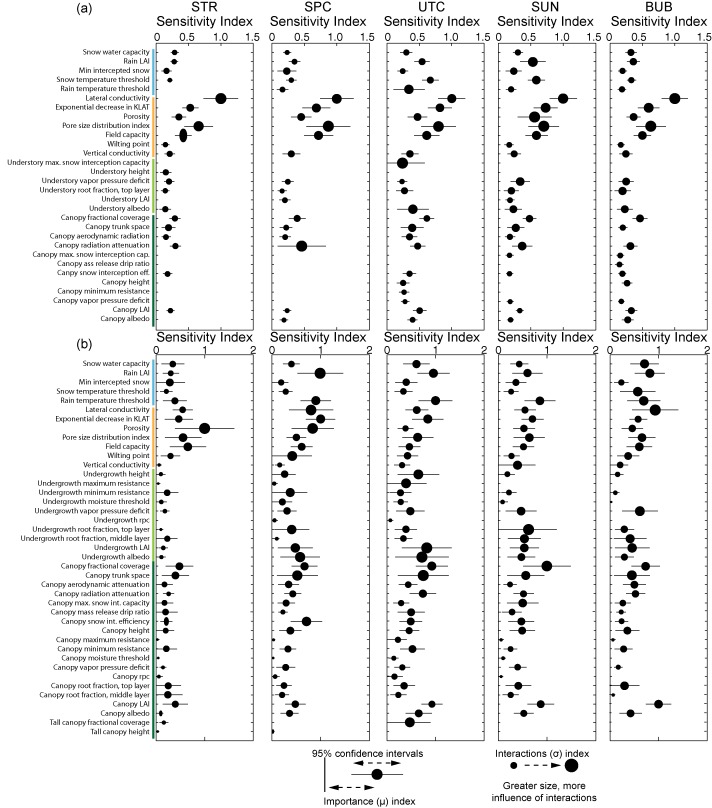
Parameter sensitivities to (a) magnitude of peak runoff and (b) timing of peak runoff for streamflow across the subcatchments. Abbreviations for subcatchments are STR, Stringer Creek; SPC, Spring Park Creek; UTC, Upper Tenderfoot Creek; SUN, Sun Creek; and BUB, Bubbling Creek. The colors of the abbreviations correspond to the results for each subcatchment.

**Figure 7:**
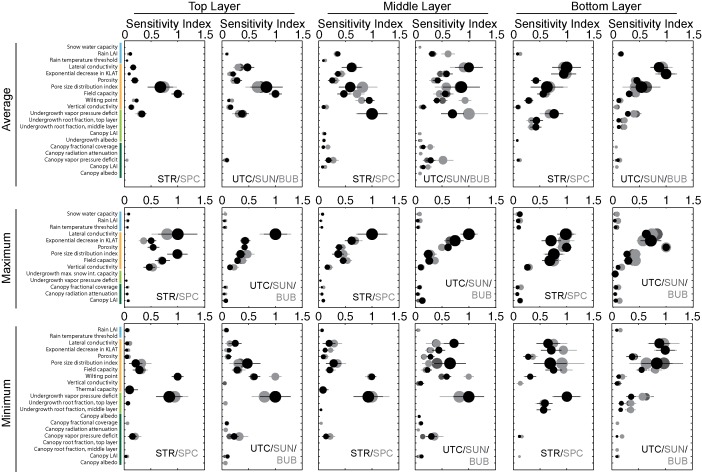
Parameter sensitivities to average, minimum, and maximum soil moisture for the (top) upper most, middle, and (bottom) deepest soil layers. Abbreviations for subcatchments are STR, Stringer Creek; SPC, Spring Park Creek; UTC, Upper Tenderfoot Creek; SUN, Sun Creek; and BUB, Bubbling Creek. The colors of the abbreviations correspond to the results for each subcatchment.

**Figure 8:**
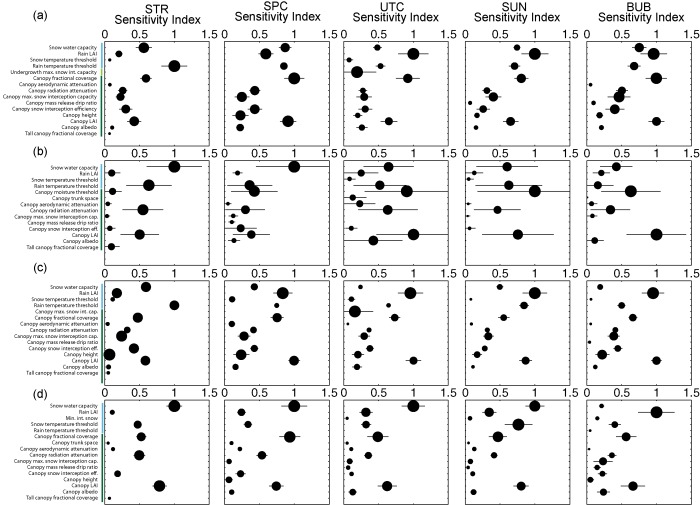
Controls on (a) peak snow water equivalent, (b) timing of maximum snow water equivalent, (c) snow water equivalent storage, and (d) maximum 3 day melt. Abbreviations for subcatchments are STR, Stringer Creek; SPC, Spring Park Creek; UTC, Upper Tenderfoot Creek; SUN, Sun Creek; and BUB, Bubbling Creek. The colors of the abbreviations correspond to the results for each subcatchment.

The identification of important parameters was performed using the mean and confidence intervals for the *μ* importance metric (i.e., average/total sensitivity), as the sensitivity index for *μ* was generally well correlated with the index for *σ*. The actual values of the sensitivity indices are unimportant, as the Method of Morris is a rank‐based sensitivity index. The values were only used to determine whether rank is distinguishable based on confidence intervals, and to determine the point at which the parameter elementary effects converge toward zero. The results for each metric and for each subcatchment were plotted, and the number of distinguishable parameters was determined via visual inspection. Only the parameters that were indistinguishable from each other and from zero were reported. We opted for this approach instead of reporting the top ranked parameters based on an arbitrary cutoff. With a screening sensitivity metric such as the Method of Morris, the meaning associated with the value of the index is not in its absolute magnitude, but its magnitude relative to the values for other parameters. As such, indices are presented as normalized to the maximum *μ* or *σ* value per subcatchment and per metric. Figures 4, 5, 6, 7, 8 display sensitivity indices across the different metrics and are organized by the flux or storage that each metric is calculated from.

### 5.3 Controls on Evapotranspiration

The results for total annual and growing season evapotranspiration are shown in Figures 4a and 4b, respectively. Annual evapotranspiration for all subcatchments was most sensitive to parameters associated with the snow model and with the vegetation characteristics. In Stringer, Spring Park, Sun, and Bubbling, there were three distinct groups of sensitive parameters. The rain temperature threshold was most important, showing the largest mean index by far and a correspondingly large *σ* index as well, indicated by the circle size across all sensitivity figures. The value of *μ* was largely correlated with the *σ* value across a majority of metrics and parameters, indicating that the parameters that most influence model output were influencing it through interactions with other parameters. The second most important parameter was the canopy fractional coverage, also with a high interactions index. Other snow and canopy parameters were also sensitive, but to a lesser extent. Sensitivity indices for Upper Tenderfoot Creek were similar, with the rain temperature threshold and the canopy fractional coverage both equally important.

Growing season evapotranspiration was sensitive to a similar but differently ordered subset of snow and vegetation parameters across all subcatchments. There was agreement across all five subcatchments in terms of overall importance, with the rain threshold again having the largest *μ* value. All subcatchments were sensitive secondarily to the same group of parameters, though with slightly different patterns of importance. The rain temperature threshold, canopy LAI, and canopy fractional coverage were all similarly dominant across all the subcatchments with the exception of Stringer Creek. Within Stringer Creek, the rain temperature threshold was most important, followed by canopy LAI. All subcatchments were similar to a similar group of secondarily important parameters, including the snow water content and other canopy parameters related to snow interception and the energy balance.

### 5.4 Controls on Streamflow

Sensitivity of parameters to streamflow across the subcatchments was assessed using a number of different metrics, to capture the behavior of high flows, low flows, timing, and flow variability. Results for the streamflow metrics are shown in Figures 5 and 6. Figure 5 summarizes sensitive parameters that describe the catchment water balance (runoff ratio), variability (coefficient of variation), low flows (base flow index), and intermediate/transition flows (slope of the flow duration curve). For three of the four metrics (runoff ratio, base flow index, and coefficient of variation), the pore size distribution index had both the largest importance and interactions index, but also the widest confidence intervals. Secondarily important values for these three metrics included a subset of snow (rain LAI), soil (lateral conductivity and its change with depth, porosity, and field capacity), and vegetation (undergrowth vapor pressure deficit) parameters. Sensitive vegetation parameters differed across the metrics with canopy parameters influencing the runoff ratio and undergrowth root fractions influencing the base flow index. Some differences in sensitive parameters existed across the metrics but were usually for parameters with lower importance indices (e.g., the importance of canopy LAI, Figures 5a, 5b, and 5d).

In contrast to results for these three metrics, where a single parameter dominated, multiple parameters had high sensitivities to the slope of the flow duration (Figure 5b). Soil parameters largely controlled response, with the exponential decrease in lateral conductivity with depth and the pore size distribution index having the largest *μ* and *σ* values for all subcatchments. Secondarily sensitive parameters across all subcatchments included soil lateral conductivity and field capacity. The lowest (tertiary) class for both subcatchment groups was similar, including a large number of parameters that span snow, soil, undergrowth, and canopy parameters.

Patterns of sensitivity to peak streamflow and timing of peak streamflow, shown in Figure 6, were highly variable across the subcatchments, with individual plots displayed for each. These subdaily metrics, in contrast to the seasonal metrics shown in Figure 5, were sensitive to a wider array of parameters. The magnitude of peak streamflow (Figure 5a) was most sensitive to soil lateral conductivity for all subcatchments. Bubbling, Stringer, and Spring Park were secondarily sensitive to the exponential decrease in lateral conductivity, porosity, pore size distribution index, and the field capacity. In addition to these parameters, Upper Tenderfoot Creek and Sun were also secondarily sensitive to the rain LAI and the snow temperature threshold. There was much overlap between parameter confidence intervals as well as high *σ* values across the subcatchments, indicating that many of the parameters are likely interacting. This was even more true for parameter sensitivities to timing of the streamflow peak. Wide confidence intervals across all subcatchments, spanning snow, soil, undergrowth, and canopy vegetation parameters render sensitivity indices largely indecipherable in terms of importance. As compared to other metrics, interactions indices for sensitivity to peak streamflow were large across all sub‐catchments, indicating that the prediction of streamflow timing is heavily dominated by parameter interactions.

### 5.5 Controls on Soil Moisture

Soil moisture response is shown for the top, middle, and bottom soil layers, with metrics for each layer corresponding to average annual soil moisture, the annual minimum, and the annual maximum values. Responses across the subcatchments were generally similar for similarly oriented subcatchments: first, between Stringer Creek (STR) and Spring Park Creek (SPC) and second, among Upper Tenderfoot Creek (UTC), Sun Creek (SUN), and Bubbling Creek (BUB). Sensitivities are displayed in these subcatchment groups in Figure 7. Average soil moisture in all layers and for all subcatchments was primarily controlled by soil parameters, but the parameters themselves varied between the layers. Average conditions were controlled by the pore size distribution index and the field capacity in the top layer and lateral conductivity and its change with depth in the bottom layer. In the middle layer, average conditions for Stringer and Spring Park were most sensitive to undergrowth vapor pressure deficit and the wilting point, and lateral conductivity, the pore size distribution index, and the undergrowth vapor pressure deficit for Upper Tenderfoot, Sun, and Bubbling. Across all layers, the pore size distribution index had a high interactions index. Interaction indices were similarly large in the bottom and middle layers for lateral conductivity, and in the middle layer for the vapor pressure deficit.

Maximum soil moisture represents saturated conditions across the subcatchments and was most sensitive to soil parameters which control the saturated and unsaturated hydraulic conductivity, with secondary sensitivity to snow and vegetation parameters. In the top layer, the highest importance and highest interactions values were associated with lateral conductivity and additionally the pore size distribution index for Stringer and Spring Park and lateral conductivity alone for the other three subcatchments. In the middle layer, the highest importance and interactions were associated with lateral conductivity and secondarily its decrease with depth. In the bottom layer, porosity was most important, followed by lateral conductivity and its decrease with depth. Across all subcatchments, snow and vegetation parameters had the lowest sensitivities. Lateral conductivity was responsible for interactions for the maximum soil moisture within all layers.

Patterns were most variable between subcatchments and layers for parameter sensitivities with respect to minimum soil moisture. While the top and middle layers were most sensitive to vegetation influence, responses in the bottom layers were also controlled by soil parameters. In the upper soil layer, the undergrowth vapor pressure deficit and the wilting point, with the former having a high *σ* index, were most sensitive to subcatchment conditions at low soil moisture. The same is true in the middle soil layer, though other soil parameters exhibited higher secondary importance indices in Upper Tenderfoot, Sun, and Bubbling. In the deepest soil layer, controls diverged. Field capacity, the pore size distribution index, and lateral conductivity and its decrease with depth were most sensitive across all subcatchments, while the undergrowth vapor pressure deficit was equally important in Stringer and Spring Park.

### 5.6 Controls on Snow Water Equivalent

We assessed controls on snow water equivalent in terms of four different metrics related to the maximum (Figure 7a), timing of maximum (and therefore start of melt; Figure 7b), total storage (Figure 7c), and melt magnitude of the snowpack (Figure 7d), calculated from subcatchment average snow water equivalent and melt rates. Overall, parameter sensitivities to the SWE metrics had narrow confidence intervals, with the exception of parameter sensitivities calculated for the timing of maximum SWE (Figure 7b), and high *σ* values, suggesting high interactions between parameters. Across all metrics and subcatchments, high parameter sensitivities corresponded to snow/climate parameters (including the snow water capacity, the rain leaf area index, and the snow and rain threshold temperatures) followed by canopy vegetation parameters. However, as compared to other metrics, we saw the greatest variability in parameter importance between subcatchments. Though the same subset of parameters was sensitive across all subcatchments and metrics, the ordering of parameters differed. Maximum SWE was most influenced by the rain temperature threshold for Stringer and Sun, the rain LAI and the canopy fractional cover for Upper Tenderfoot Creek, canopy fractional cover for Spring Park, and the rain LAI, canopy fractional cover, and canopy LAI for Bubbling. Similar patterns were observed for total storage (Figure 7c), the differences being that canopy LAI played a larger role in Spring Park, Upper Tenderfoot, and Sun and maintained importance in Bubbling Creek, and the rain LAI was important for all basins with the exception of Stringer Creek. Melt was most influenced by the snow water capacity for all subcatchments but Bubbling, which was most influenced by the rain LAI. Parameter sensitivities with respect to timing of maximum SWE all had very wide confidence intervals, similar to timing of peak streamflow, rendering deciphering key parameter influences quite difficult. The most sensitive parameters, which also had the widest confidence intervals, were some combination of snow water capacity, the rain temperature threshold, canopy radiation attenuation, and canopy LAI across all subcatchments. Secondary controls across all metrics included some subset related to fractional canopy coverage, canopy LAI, and snow water capacity. While sensitive parameters were largely similar across the subcatchments, the snow metrics were sensitive to a different subset of parameters than those identified for evapotranspiration, streamflow, and soil moisture. These parameters were largely canopy parameters related to accumulation and melt.

## 6 Discussion

Given our limited understanding of headwater catchments and the lack of data within these areas, we need new approaches to headwater hydrology that interrogate these parts of the landscape even in the absence of observations. Here we develop a comparative hydrology framework that uses global sensitivity analysis across a priori parameter ranges to identify parameters that most influence hydrologic partitioning across five adjacent headwater subcatchments. Parameter sensitivities are assessed with respect to metrics calculated from model‐predicted hydrologic behavior, a difference as compared to many modeling studies that assess sensitivity with respect to error metrics. This fundamental difference has great potential for detecting dominant landscape influences on hydrology in ungauged headwater catchments, as no data are required. To test whether this approach is acceptable, we compare our results to a range of literature from Tenderfoot Creek that has assessed landscape controls on hydrologic behavior. We ask both whether sensitive parameters detected through this analysis make “process‐sense” when compared with our understanding of catchment function, and how variability in these sensitivities across subcatchments either supports or refutes our understanding of similarities and differences in subcatchment topography and vegetation.

### 6.1 DHSVM Parametric Controls on TCEF Hydrologic Dynamics

**Figure 9:**
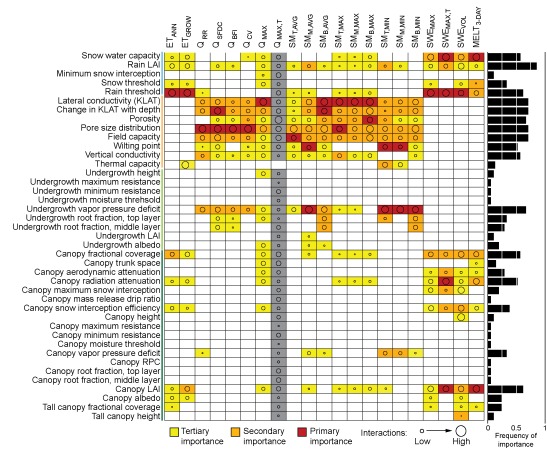
Summary of primary, secondary, and tertiary importance sensitivity indices for Stringer Creek. Color indicates importance; gray boxes correspond to parameter sensitivity indices with high uncertainty. Circle size indicates the degree of interactions.

**Figure 10:**
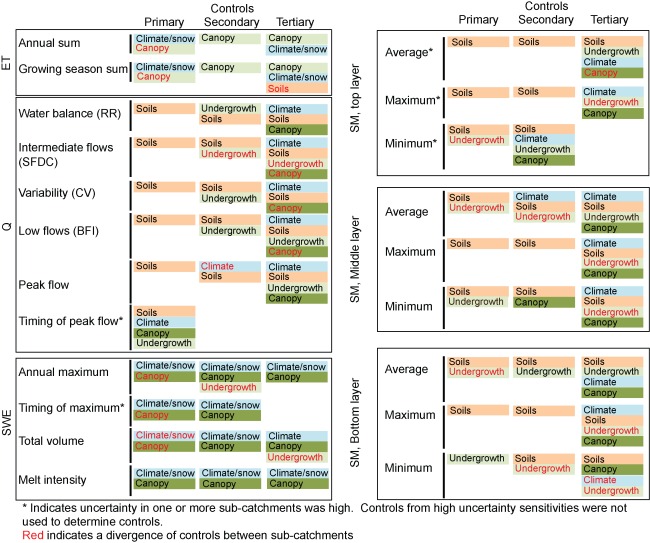
Summary of the parameter types (snow, canopy, undergrowth, and soil) that are primary, secondary, or tertiary controls on model output metrics.

Interestingly, the model‐based sensitivity analysis identified a similar group of sensitive parameters across all subcatchments and all metrics. This is in spite of physical variability incorporated as model input between subcatchments. Given the similarities in parameter sensitivities between the subcatchments, we summarized sensitive parameters for representative subcatchment Stringer Creek (Figure 9) and point out differences across subcatchments where appropriate. Parameter importance for each metric was visually classified as primary, secondary, or tertiary based on relative values of sensitivity and confidence interval widths. Results were generalized across the subcatchments (Figure 10).

When summarized, patterns are evident across the metrics and subcatchments. Though differences exist among individual metrics, streamflow (Figures 5 and 6) and soil moisture (Figure 7) metrics were most sensitive to soil and snow parameters, while evapotranspiration (Figure 4) and snow water equivalent (Figure 8) metrics were entirely sensitive to snow and canopy vegetation parameters. In contrast to soil or vegetation parameters, many of the snow parameters were sensitive across all metrics. While this seems intuitive given that Tenderfoot Creek is a snow‐dominated system, it also suggests that the snow input to the catchment is one of the primary constraints on hydrologic partitioning to evapotranspiration, streamflow, and soil moisture.

Model‐predicted evapotranspiration (ET; Figure 4) and snow water equivalent (Figure 8) were controlled by a very similar set of model snow and canopy vegetation parameters. Interestingly, both annual and growing season evapotranspiration were most influenced by climate and the separation between rain and snow (in terms of the rain temperature threshold) while growing season evapotranspiration was additionally influenced by leaf area index and canopy fractional coverage, both important due to their impacts on the magnitude of interception and rates of potential evapotranspiration. These same canopy and snow/climate parameters related to the potential and actual calculation of evapotranspiration are also related to the retention of snow and subsequent melt. Thus, the same parameters that are most sensitive to the prediction of ET are also sensitive to the prediction of SWE. Vegetation, especially canopy vegetation, is used within the routines that determine snowmelt and therefore the distribution of water across the subcatchments. Additionally sensitive parameters to SWE metrics alone included overstory presence, height, and other characteristics controlling aerodynamic resistance and snow water accumulation and melt (snow water capacity and snow temperature threshold). From a physical perspective, the importance of vegetation density to snow accumulation and melt also fits with our broad understanding of Tenderfoot Creek hydrology [*Woods et al*., 2006]. Other studies have shown that snow accumulation and melt can be dependent on the density of vegetation within a catchment, and that thinning might impact the magnitude and timing of spring snowmelt [*Ellis et al*., 2013]. Parameter sensitivity to SWE, while not extremely variable across metrics, was especially interesting when contrasted with controls on streamflow and soil moisture across hydrologic seasons.

Parameter sensitivities to streamflow (Figures 5 and 6) and soil moisture (Figure 7) differed with subcatchment wetness state. During dry periods, subcatchment streamflow was controlled by the pore size distribution index, which influences the lateral redistribution of soil water in the unsaturated zone. In contrast, during wet periods streamflow was most sensitive to soil lateral conductivity, which primarily influences the saturated movement of water through soil. In addition to this parameter, streamflow metrics describing wet catchment states were more sensitive to vegetation parameters, specifically the leaf area index, tree density (fractional coverage), and undergrowth height and root distribution.

This analysis of differing controls under wet versus dry catchment conditions was similarly used to interpret parameter sensitivities to soil moisture with depth. For each of the three soil moisture layers, we assessed sensitivity to minimum (dry), maximum (wet), and average (overall) soil moisture across the year, thus capturing parameter controls across a gradient of wetness states. The deepest soil layer, expected to be saturated the longest, was entirely controlled by parameters related to soil saturation (porosity) during the wettest periods and saturated moisture movement (lateral conductivity) under average and dry conditions. In contrast, the upper and middle soil layers were sensitive to saturated soil moisture movement during wet times (lateral conductivity), but to soil parameters related to plant available water (field capacity and wilting point), unsaturated moisture movement (pore size distribution index), and rates of evapotranspiration (vapor pressure deficit) during average and dry periods. In this sense, these layers were most influenced by soil properties that either facilitate or limit soil water extraction.

While we deduced differences in sensitive parameters across the metrics, a very similar subset of parameters was consistently sensitive across all metrics and all subcatchments. This suggests that local variability due to topography and vegetation may lead to small differences in subcatchment characteristics and therefore parameter sensitivities, but that sensitive parameters were generally the same given similar seasonal and annual climate across Tenderfoot Creek. Local climatic variability at smaller temporal scales and across Tenderfoot Creek subcatchments was minimal and primarily impacted by topography. The subcatchments span 441 m of elevation range, with 263 m of this distance captured by the two SNOTEL sites. However, the catchment area represented by high elevations outside the SNOTEL site elevation range is small.

The limited variability that we found between parameter sensitivities across the subcatchments suggests that the factor that unites these subcatchments—climate—may organize parameter sensitivities at much larger spatial scales. Climate has been shown by numerous studies to be the primary control on annual water balance [*Budyko*, 1974; *Eagleson*, 1978; *Milly*, 1994; *Williams et al*., 2012]. This has interesting implications for the extension of this method to other headwater sites. We hypothesize that the parameters that are sensitive and how sensitive they are will vary across much larger spatial scales, first as a function of climate and secondarily as a function of topography and vegetation. Small differences in sensitivities between subcatchments are likely driven by differences in topography (and therefore local deviations in climate) and vegetation types. As soil type does not vary between the subcatchments, we do not expect variability in soil properties to impact parameter variability detected here. However, for other applications where soil properties are more heterogeneous, this will likely impact parameter sensitivities when comparing collocated subcatchments. Geology is not explicitly incorporated into the modeling framework, and its impact on parameter sensitivities is therefore not testable with DHSVM.

### 6.2 Comparison to Current Perceptual Understanding of Tenderfoot Creek

In this study, we tested the extent to which a model‐based sensitivity analysis can be used in the absence of observations to improve our understanding of ungauged areas of the landscape. We found that a similar subset of model parameters is sensitive across the subcatchments, leading to the following questions:

(1) How do sensitive parameters detected through model analysis compare with our understanding of catchment function? Do they make process sense?

(2) What variability between subcatchments should we expect?

(3) How do parameter sensitivities either support or refute our empirical understanding of how these subcatchments function?

(4) Where in Tenderfoot Creek is this approach acceptable, and where is it not?

#### 6.2.1 Parameter Sensitivities Across the Subcatchment

In this approach, we qualitatively compared the results of the sensitivity analysis to our experimental understanding of expected similarities and differences in subcatchment functioning. This is an alternative to the traditional approach of testing parameter sensitivities only with respect to error metrics and serves as an evaluative step to determine the information this approach can provide in the absence of observations. Experimental understanding of subcatchment function has been derived from many empirical and model‐based investigations and comparative studies that have been performed over the last few decades. The results of this past work corroborate many of the findings presented here, and we compare our experimental perceptions versus the results of the sensitivity analysis to evaluate the veracity of our comparative hydrology approach.

Perception of controls on evapotranspiration in Tenderfoot Creek primarily emphasize the first order importance of tree height as it relates to rooting depth and leaf area index in Lodge Pole pines [*Keane et al*., 2005; *Jensen et al*., 2008; *Nippgen et al*., 2011; *Emanuel et al*., 2010]. While we found that both annual and growing season evapotranspiration were somewhat sensitive to tree height and other canopy‐related properties, we also found that parameters related to the energy budget as well as parameters related to the snow module were most important to predicting annual ET. This suggests that snow accumulation and melt were such driving forces in these catchments that the associated snow and energy parameters were more important to ET magnitudes than the vegetation properties themselves. In the absence of observations, this implies that testing for and ensuring accurate timing and magnitude of SWE would be particularly important to predicting not only soil moisture and streamflow but also annual evapotranspiration. Given this emphasis, we recommend that measurements to guide future modeling and calibration should focus on snow sampling across the season and at locations of differing elevation to capture local variability. During the driest parts of the year, we also expect differing controls on ET. Modeling by *Emanuel et al*. [2010] suggested that late summer evapotranspiration was increasingly limited by soil moisture‐related water stress. In contrast to these expectations, ET was not sensitive to soil parameters during the growing season. June and July were likely too early to detect this effect, and soil parameters may play a role later in the season (e.g., August or September).

Streamflow response in the Tenderfoot Creek subcatchments is primarily driven by snowmelt, with peaks in late May or early June, a period of dry‐down through the summer, and low flows through the winter. Despite this seasonal streamflow pattern, previously performed modeling work suggested strong connection between snow accumulation/melt and streamflow. Work by *Nippgen et al*. [2011] used a module of the Transfer Function Hydrograph Separation Model (TRANSEP) [*Weiler et al*., 2003] to determine mean runoff response time. This metric represents the average amount of time it takes for effective precipitation (precipitation and snowmelt) to exit the catchment outlet. The study compared this response across the five Tenderfoot Creek subcatchments as well as at the Tenderfoot Creek outlet and a stream gage located in the middle of Stringer Creek. *Nippgen et al*. [2011] determined that interannual variability in the mean response time was particularly influenced by the annual maximum SWE, highlighting the importance of snowmelt to the prediction of streamflow. While *Nippgen et al*. [2011] did not explicitly test the impact of soil parameters on streamflow, their conclusion that SWE magnitude influences streamflow timing corroborates our findings that many of the vegetation parameters that are sensitive to SWE metrics are also sensitive to streamflow metrics.

*Ahl et al*. [2008] applied the Soil and Water Assessment Tool (SWAT) to the Tenderfoot Creek outlet for 1997–2002. Their analysis compared a model run for calibrated parameters to a model run for model default parameters. Parameter sensitivity was assessed with respect to the Nash Sutcliffe Efficiency coefficient (NSE), which emphasizes the timing and magnitude of peak flows. With this formulation, *Ahl et al*. [2008] found that snowmelt parameters most influenced streamflow prediction. They also found soil parameters and SCS curve number parameters (representative of land cover/vegetation) had minimal effect on model efficiency. This is opposite of our findings for parameters that were sensitive to the timing of peak streamflow but aligned with our findings for the magnitude of peak streamflow. While we arrived at similar conclusions for one of the metrics, the disagreement over the importance of soil parameters is likely due to differences in the model formulation as well as differences in the analysis. Specifically, the Method of Morris is a more rigorous assessment of parameter sensitivity than the one‐at‐a‐time sensitivity analysis performed by *Ahl et al*. [2008].

In another modeling study of Tenderfoot Creek, *Emanuel et al*. [2010] concluded that temporal influences on water stress within the Stringer Creek subcatchment varied across the growing season. Through their application of a spatially distributed model, the Soil‐Vegetation‐Atmosphere Transfer model, they found that evapotranspiration became decoupled from vapor pressure deficit during increasingly drier times in late summer. During this drier period, ET was instead most influenced by the availability of soil water. As such, we expected soil moisture, especially shallow soil layers during drier periods, to be influenced not only by unsaturated flow processes but also by vegetation. We also expected deeper soil layers during snowmelt to be most influenced by properties that control the lateral redistribution of water. Overall, patterns of sensitive parameters for wet times and dry times followed these expectations. Parameter influence on average soil moisture conditions varied with depth, with the role of vegetation being greater for shallower layers during drier times and the role of lateral water redistribution remaining more important in deeper soil layers (Figure 7).

Some differences emerged in terms of which parameters we would expect to influence hydrologic behavior across Tenderfoot Creek subcatchments. However, the overall subset of important parameters (Figure 9) did not exclude any parameters we would expect to be sensitive. Given our findings, it is worth investigating how parameter influences on hydrologic partitioning will vary across other climates, topographies, and vegetative covers. Future work should address the spatial scale, in terms of variability in climate, and the variability in soil and vegetation type and distribution at which parameter sensitivities could begin to diverge for other catchments.

#### 6.2.2 Similarities and Differences in Parameter Sensitivities Between the Subcatchments

We observed similarities in parameter sensitivities between subcatchments. However, we are also interested in whether we could detect differences in parameter sensitivities due to subcatchment variability in topography and vegetation. Our ability to identify differences in parameter sensitivities between subcatchments could provide insight into transferability of model results and parameter sensitivities. Past studies have explained differences in runoff dynamics across Tenderfoot Creek subcatchments in terms of vegetation, geology, and topography [*Nippgen et al*., 2011; *Jencso and McGlynn*, 2011]. Both *Nippgen et al*. [2011] and *Jencso and McGlynn* [2011] found variable hydrologic behavior across the Tenderfoot Creek subcatchments, particularly with previously clear‐cut (Sun Creek and Spring Park Creek) subcatchments behaving differently than the others.

In this study, we were unable to find strong differences in controls between the subcatchments: important parameters (Figures 4, 5, 6, 7, 8) were more similar than they were different. Many catchment level differences are incorporated directly into the DHSVM model structure (e.g., topography and distribution of vegetation), while others are not explicitly included (e.g., geology). With regards to variation in vegetation, the study area includes two sets of paired catchments, Stringer Creek/Spring Park Creek and Bubbling Creek/Sun Creek. Within these sets, the subcatchments have similar aspect and elevation but different distributions of canopy versus undergrowth vegetation. This amounts to 15% more canopy vegetation in Stringer Creek as compared to Spring Park and about 24% more canopy vegetation in Bubbling Creek as compared to Sun Creek. Despite this, all metrics across these subcatchments were sensitive to similar parameters. This suggests that even in the face of partial forest harvest, parameter importance to hydrologic behavior may not change. While this type of disturbance might not impact parameter sensitivities, it may likely be reflected in differences in vegetation parameter values.

The largest differences in parameter sensitivities between the subcatchments occurred with respect to snow metrics. As we expect predictions of snow accumulation and melt to be most influenced by the two spatially distributed inputs that vary the most between the catchments—vegetation distribution and topography—the fact that we do see small differences in the organization of parameter sensitivities does fit with our understanding of Tenderfoot Creek as well as the general functioning of snow‐dominated catchments. While vegetation cover likely plays a role, we primarily conclude that small differences in parameter sensitivities between the subcatchments are due to differences in watershed slope and structure. Given the small spatial scale (22.5 km^2^) of Tenderfoot Creek and its modest elevation gain (∼350 m), we assert that climatic inputs and land‐atmosphere transfers were largely consistent across the subcatchments. As such, we infer that observed variability in hydrologic response was most likely due to subcatchment structure, a conclusion consistent with past empirical data analysis and observations. Previous research in Tenderfoot Creek suggests that topographic structure is likely the largest source of variability driving differences in hydrologic partitioning across the subcatchments [*Nippgen et al*., 2011; *Jencso et al*., 2010]. As topographic structure is a prescribed input, we could not explicitly test its effect within the sensitivity analysis. However, we observed differences in runoff behavior with only modest variability in model parameter sensitivity across the subcatchments.

#### 6.2.3 Where in Tenderfoot Creek Is This Approach Acceptable, and Where Is It Not?

There are three aspects of this sensitivity analysis that are unique when compared to sensitivity analyses performed for other complex, distributed models. First, we perform an analysis that takes into account not only parameter importance but also interactions. Second, we examine response with regard to metrics beyond streamflow, considering parameter influences on a range of hydrologic behavior. Third, we only investigate model‐predicted behavior. This last point is perhaps the most important, given that data and especially time‐varying measurements for model calibration are often the limiting factor for the application of these types of models to headwater streams. Our goal here was to perform a sensitivity analysis in the absence of point measurements, to assess the amount of information that can be obtained from an analysis of a priori data, expert opinion, and state‐level/global data sets.

It is also worth noting that sensitivity analyses are often performed with respect to objective functions and may use fuzzy metrics [e.g., *Pappenberger and Beven*, 2004; *Kelleher et al*., 2013] to distinguish between behavioral and nonbehavioral parameter sets (those which represent observed behavior to some level of acceptability). We instead chose to include all model runs in our sensitivity analysis, opting to add little to no field information to our analysis as this approach is meant to be applied in ungauged areas where such information would not be available. Introducing a fuzzy metric even in the absence of observations is still possible but would likely be best applied after a screening sensitivity analysis like the Method of Morris has reduced the size of the parameter space.

We recognize that the assumption we make in the introduction—*that the model is a good mechanistic representation of catchment processes*—may not be true everywhere. While error metrics would be one way to address this assumption, calculation of error metrics requires the presence of observations. As an alternative for ungauged catchments, we assert that sensitivity index confidence intervals may also be used to detect parts of the landscape where the model is either a good or a poor representation of catchment behavior.

Initial analysis with DHSVM applied to Tenderfoot Creek subcatchments was performed with a uniform solar radiation forcing, varied by a modifier that we included as a model parameter. While sensitivity results featured narrow confidence intervals for a majority of the subcatchments, we found consistently wide confidence intervals for one subcatchment, Upper Tenderfoot Creek (e.g., Figures 4a, 6a, and 8b). Within this area, uncertainty in the detection of parameter controls indicated either a problem with inputs (errors in meteorological inputs or their distribution, inaccurate or imprecise a priori parameter ranges, or misrepresented or lack of heterogeneity in spatial inputs) or that an important catchment process is absent in the model. The latter was the most plausible for Upper Tenderfoot Creek, and reassessing parameter sensitivities with the distribution of solar radiation produced sensitivities with narrower confidence intervals. Interestingly, parameter sensitivities for the other subcatchments were largely similar whether the model was forced with uniform or spatially variable solar radiation (see supporting information). This suggests that forcing the subcatchments that have a dominant, as opposed to a mix (like Upper Tenderfoot Creek), of aspects has potential to capture key annual and seasonal controls. However, the same may not be true at finer timescales. Interestingly, when forcing the model with spatially variable solar radiation, we found our confidence intervals for the timing of both peak streamflow and peak SWE grew wider. This was likely because the input of solar radiation is such a driving force in these subcatchments that, when represented by a model parameter, it subsumed the majority of sensitivity. With the implementation of spatially variable solar radiation and subsequent removal of this solar radiation modifier, results in Figures 6 and 8 highlight that these finer temporal scale metrics are influenced by a range of catchment characteristics and entirely driven by parameter interactions, as we would expect for as complex a mechanism as the timing of melt or peak streamflow occurrence.

One potential limitation of this application is that the role of geology is not currently included in the DHSVM model framework but is known to be an important control on streamflow generation within this subcatchment [*Jencso and McGlynn*, 2011; *Payn et al*., 2012]. While empirical and modeling studies have highlighted the general correlation between geology and streamflow [*Nippgen et al*., 2011] and water table [*Jencso and McGlynn*, 2011] response across Tenderfoot Creek, recent work suggests the presence of a fault line cutting through parts of Upper Tenderfoot Creek, Sun Creek, and minimally Bubbling Creek that may impact hydrologic behavior [*Payn et al*., 2012]. Additionally, a region located in Upper Tenderfoot Creek, “Onion Park,” has been shown to be a major source of additional water to the stream due to its association with a Quartzite ridge along the eastern edge of the Tenderfoot Creek catchment [*Pacific et al*., 2010; *Payn et al*., 2012]. Given this, any future model calibration may need to incorporate the role of geology into the model framework. If DHSVM is not able to accurately predict streamflow following model calibration, one may need to utilize another model with a geologic/deep‐groundwater framework [e.g., RHESSys; *Band et al*., 1991, 1993; *Tague and Band*, 2004].

Given DHSVM's preexisting model framework, it is likely this model is best used in areas where geology does not play an important role in hydrologic processes. Additionally, since the model lacks a full dynamic infiltration module, it is also best applied in areas where infiltration does not control runoff generation, and should be tested in flat areas that are both very wet and very dry to ensure that the absence of dynamic infiltration routines does not impact model fit. Additional applications should also consider the level of detail required to distribute meteorological inputs across each catchment.

### 6.3 What are the Implications of This Study for Application to Other (Ungauged) Catchments?

Applying a physically based model to an ungauged catchment does not necessarily require observations but does necessitate reasonable perception of how the catchment functions. In the case of the Tenderfoot Creek Experimental Forest, this perception is supported by a 20+ year record of climate and 10+ year record of streamflow, as well as hundreds of thousands of water table measurements and many field seasons of observations [*Jencso et al*., 2009, 2010; *Pacific et al*., 2010; *Emanuel et al*., 2010; *Nippgen et al*., 2011; *Jencso and McGlynn*, 2011]. However, we argue that a reasonable understanding of a system can be constructed from limited observations. As described in terms of the information hierarchy by *McGlynn et al*. [2013], information from global and national data sets, to local field visits, to dedicated measurements can help to refine an understanding of important hydrological processes and what governs them. Global and national data sets can inform perception via information about annual and seasonal climatology as well as physical characteristics of the catchment (eco‐regions, hydrography, and soils). However, global and national data sets can miss local catchment variability that can govern dominant processes surface partitioning and internal catchment water redistribution that results in runoff generation. Local field visits and/or expert judgment can add great value to perception of catchment functioning. Gathering local information such as bedrock geology type (primary and secondary porosity), soil depths, location and type of vegetation on the landscape, and observations of signs of overland flow or lack thereof will further improve an understanding of the timing and magnitudes of hydrologic partitioning at subsequently finer spatial and temporal scales.

As exemplified by Tenderfoot Creek subcatchments, there are parts of the landscape where defining and improving perception of dominant processes and the characteristics that define process magnitudes and time scales can take much more effort. This type of approach has the potential to highlight parts of the catchment where perceptions and mechanistically derived sensitivities diverge, suggesting that some process unobserved on the landscape may be influencing hydrologic behavior. For catchments where this approach results in wide confidence intervals, this may signify the need for observations of streamflow at high temporal resolution, as a dominant runoff generation mechanism or partitioning process that may be unobservable from data sets or pictures alone (e.g., a fault line and a spring). Constraining subsurface behavior can be particularly difficult without higher‐level observations, especially depending on the coarseness of soil surveys with depth or in space (e.g., confining soil layers with minimal thickness and variability of soil depth in space). Catchments in altered landscapes also likely require more information than natural catchments in order to identify important catchment characteristics and hydrologic processes in space and time. There is still limited understanding of how many landscape‐level changes affect the hydrology of a given catchment in a given climate, and even more limited understanding of how this varies at different spatial and temporal scales. Hypothesizing governing influences on hydrologic processes at smaller spatial and temporal scales however can be improved by adding information, either in the form of local site visits/expert judgment or field measurements, when possible. In these cases, evaluating perceptions of catchment functioning against a physically based model may help to guide investigations and can highlight periods of the year or parts of the landscape where perception and the mechanistic model diverge, therefore identifying specific times or locations that require further investigation for field campaigns or greater caution due to elevated uncertainty.

Given that we can infer dominant hydrologic behaviors and the catchment characteristics that govern them from limited information, we propose that the approach described here is a useful method for identifying key catchment characteristics and their influence on hydrologic behavior. We also suggest this approach can be used to test hypotheses regarding the relationships between hydrologic processes and catchment characteristics. In this approach, researchers would construct physically based models of catchments from a combination of global and national data sets as well as local judgment/measurements when available. The modeling requirements for this type of application are similar to the requirements for identifying dominant catchment processes: spatially distributed soil, vegetation, topography, climate forcing, and stream channel locations, all of which can be obtained in the US at some resolution from national data sets. Applying a physically based model can be done with minimal dedicated measurements, as sensitivities are assessed with respect to model‐predicted states and fluxes, not error metrics. Parameter bounds are constrained a priori, and bounds can be obtained from national and global data sets for vegetation and soil information. When detailed soil surveys or vegetation species type are not available, plant and soil parameters can be generalized based on regional information.

While it may not be difficult to create a model framework, the modeler must remember that there are likely to be abstractions or missing pieces in either the system perception or the physically based model, or in some cases both. Thus, evaluating what can be learned from this type of application should always consider the level of uncertainty the modeler and/or catchment expert has in these tools, and where they need to be refined. While implementing this approach in catchments with minimal observations has the potential to identify many areas where we need to advance our understanding of hydrology, it may also be useful for highlighting large parts of the landscape where our understanding is solidly rooted in the observations already present in the record.

## 7 Conclusions

Headwater streams are widely uninstrumented but incredibly abundant. Thus, to advance our understanding of these systems and to represent them with models, we need approaches that allow us to learn about headwater catchments but do not require point measurements of streamflow or other hydrologic processes. To address this need, we have developed and tested a comparative hydrology approach within a modeling framework, using a mechanistic model combined with global sensitivity analysis to identify dominant controls on model‐predicted hydrologic partitioning. This analysis was performed across several adjacent but differing headwater subcatchments to understand how parameter sensitivities are impacted by similarities and differences across variable landscape settings. Despite differences in vegetation distributions and topography, parameter sensitivities across subcatchments were similar in space (between subcatchments) and time (between metrics). While sensitive parameters for snow water equivalent and evapotranspiration metrics were similar across all metrics, parameters sensitive to streamflow and soil moisture metrics differed across the subcatchments depending on the wetness state of the subcatchment or soil layer.

Overall, a similar subset of parameters were identified as sensitive for metrics describing hydrologic partitioning across a number of subcatchment states, suggesting that the number of model factors that most influence hydrologic output is only a subset of parameters available in a complex model such as DHSVM. However, we hypothesize that the model parameters that will most influence a given catchment will differ first with climate and secondarily with topography and vegetation cover. This work represents an unprecedented and rigorous sensitivity analysis of DHSVM across multiple headwater subcatchments, considering multiple model states and fluxes under a range of hydrologic conditions. Previous sensitivity analyses of DHSVM have applied one‐at‐a‐time sensitivity analyses at annual time scales [*Cuo et al*., 2011]. Here we quantified the influence of parameter interactions as well as investigated parameter sensitivities with respect to hydrologically relevant metrics at annual to subannual time scales.

Confronting perceptions of dominant hydrologic processes with physically based models has potential to improve our representation of headwater processes as well as our understanding of catchment functioning. Here our application focused on the assessment of multiple metrics that represent hydrologic partitioning across catchments, as well as a priori and expert‐constrained parameter bounds. Results were also evaluated against expert knowledge and perception of catchment functioning [e.g., *Emanuel et al*., 2010; *Nippgen et al*., 2011; *Jencso and McGlynn*, 2011]. The assessment of these metrics is a first step toward improving model applications in headwater systems. However, its focus on subcatchment average metrics neglects expected spatial variability in hydrologic partitioning that should be investigated in future model calibration studies. For example, parameter sensitivities have been shown to differ spatially for much larger catchments (e.g., catchment size of 1248 km^2^) [*van Werkhoven et al*., 2008; *Herman et al*., 2013], indicating that catchment simulations are subject not only to temporal but also to spatial parameter equifinality. Given that Tenderfoot Creek researchers have found significant spatial variability in hydrologic partitioning [*Emanuel et al*., 2010; *Jencso and McGlynn*, 2011], accurate, predictive modeling of headwater catchments should ultimately consider how parameters represent important processes not only in time but also in space.

Though our approach was initially tested in a set of catchments with extensive measurements, we contend that this is not required. As an alternative to extensive monitoring, we suggest instead that a strong understanding of catchment processes and the characteristics that govern them can be used to generate and test parameter sensitivities for a physically based model. The model output and parameter sensitivities can then be assessed against these perceptions, to evaluate and refine our understanding of how a given catchment functions and more broadly to understand dominant controls across variable climate, topography, and vegetation. Evaluating model‐predicted outputs requires a strong perception of system processes that would likely require more information than what can be derived from global and national data products. Site characteristics and catchment‐specific knowledge could then help refine a perceptual model for a region into a perceptual model for a place. Ultimately, this type of framework would improve our understanding of similarities and differences in hydrologic behavior across headwater streams and could assist researchers to more broadly understand how relationships among soil, vegetation, and eventually climate may influence hydrologic behavior.

## Supporting information

Supporting Information S1
