# Supplementary material for: Model‐based analysis of the influence of catchment properties on hydrologic partitioning across five mountain headwater subcatchments
Source: Water Resour Res. 2015 Jun 9;51(6):4109–36. doi: 10.1002/2014WR016147 (PMC5008156; doi:10.1002/2014WR016147)
Supplement: Supplementary file 1 — Supporting Information S1 [file WRCR-51-4109-s001.doc]

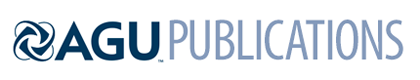


*Water Resources Research*

Supporting Information for

**Model-based analysis of the influence of catchment properties on hydrologic partitioning across five mountain headwater sub-catchments**

Christa Kelleher^1^, Thorsten Wagener^2^, Brian McGlynn^1^

1. Department of Earth and Ocean Sciences, Nicholas School of the Environment, Duke University, Durham, NC.

2. Department of Civil Engineering, Bristol University, Bristol, UK

**Contents of this file**

Text S1 to S5

Figures S1 to S14

Tables S1 to S35

**Introduction**

The following displays tables of Morris sensitivity indices and figures for Morris results for the five sub-catchments for all metrics. Content is organized by metrics and model fluxes/storages. Table and figure indices have been normalized in the text, but exact values for each parameter and each metric are contained here.

Parameters in tables are indicated by type (T): basin snow/energy (B), soil (S), undergrowth (U), lodgepole pine (O), and tall canopy trees (T). Columns are organized by metrics. For each metric:

The first column displays the mean sensitivity index (μ)

The second column displays the 95% confidence intervals for the sensitivity index (±CI)

The third column displays the interactions sensitivity index (σ), and

The fourth column displays the 95% confidence intervals for the sensitivity index (±CI).

Figures display all indices across sub-catchments, and plot the interactions index (σ, x-axis) against the mean index (μ, y-axis). Non-negligible parameters are colored in black while others are shown in light grey. Numbers next to parameters in figures reference the parameter numbers shown in tables. Numbers are used to label non-negligible parameters.

We also include sensitivity analysis results forced with a uniform solar radiation modifier. Figures S8 to S14, in the same format as Figures 4 through 10, illustrate sensitivity results for all parameters and an additional solar radiation modifier parameter, used to vary solar radiation uniformly across each sub-catchment.

Text S1.

Morris results are shown for evapotranspiration metrics (annual sum and growing season sum) for Stringer Creek (Table S1), Spring Park Creek (Table S2), Upper Tenderfoot Creek (Table S3), Sun Creek (Table S4), and Bubbling Creek (Table S5). Indices are shown for all sub-catchments in Figure S1.

Table S1. Morris indices and 95% confidence intervals for evapotranspiration for Stringer Creek. Columns are organized by metrics, and rows are organized by parameters.

Table S2. Morris indices and 95% confidence intervals for evapotranspiration for Spring Park Creek. Columns are organized by metrics, and rows are organized by parameters.

Table S3. Morris indices and 95% confidence intervals for evapotranspiration for Upper Tenderfoot Creek. Columns are organized by metrics, and rows are organized by parameters.

Table S4. Morris indices and 95% confidence intervals for evapotranspiration for Sun Creek. Columns are organized by metrics, and rows are organized by parameters.

Table S5. Morris indices and 95% confidence intervals for evapotranspiration for Bubbling Creek. Columns are organized by metrics, and rows are organized by parameters.

Figure S1. Morris indices for evapotranspiration metrics, with σ (standard deviation index) plotted versus μ (mean index) across the five sub-catchments. Numbers refer to parameters as noted in Tables above. Sub-catchment abbreviations are Stringer Creek (STR), Spring Park Creek (SPC), Upper Tenderfoot Creek (UTC), Sun Creek (SUN), and Bubbling Creek (BUB).

Text S2. Morris results for streamflow metrics.

Morris results are tabulated for runoff ratio, baseflow index and slope of the flow duration curve for Stringer Creek (Table S6), Spring Park Creek (Table S7), Upper Tenderfoot Creek (Table S8), Sun Creek (Table S9), and Bubbling Creek (Table S10). Indices are plotted against each other for each of these metrics and each sub-catchment in Figure S2.

Morris results are tabulated for the coefficient of variation and the timing and magnitude of peak streamflow for Stringer Creek (Table S11), Spring Park Creek (Table S12), Upper Tenderfoot Creek (Table S13), Sun Creek (Table S14), and Bubbling Creek (Table S15). Indices are plotted against each other for each of these metrics and each sub-catchment in Figure S3.

Table S6. Morris indices and 95% confidence intervals for runoff ratio, baseflow index, and slope of the flow duration curve for Stringer Creek. Columns are organized by metrics, and rows are organized by parameters.

Table S7. Morris indices and 95% confidence intervals for runoff ratio, baseflow index, and slope of the flow duration curve for Spring Park Creek. Columns are organized by metrics, and rows are organized by parameters.

Table S8. Morris indices and 95% confidence intervals for runoff ratio, baseflow index, and slope of the flow duration curve for Upper Tenderfoot Creek. Columns are organized by metrics, and rows are organized by parameters.

Table S9. Morris indices and 95% confidence intervals for runoff ratio, baseflow index, and slope of the flow duration curve for Sun Creek. Columns are organized by metrics, and rows are organized by parameters.

Table S10. Morris indices and 95% confidence intervals for runoff ratio, baseflow index, and slope of the flow duration curve for Bubbling Creek. Columns are organized by metrics, and rows are organized by parameters.

Figure S1. Morris indices for runoff ratio, baseflow index, and the slope of the flow duration curve, with σ (standard deviation index) plotted versus μ (mean index) across the five sub-catchments. Numbers refer to parameters as noted in Tables above. Sub-catchment abbreviations are Stringer Creek (STR), Spring Park Creek (SPC), Upper Tenderfoot Creek (UTC), Sun Creek (SUN), and Bubbling Creek (BUB).

Table S11. Morris indices and 95% confidence intervals for the coefficient of variation and the timing and magnitude of peak streamflow for Stringer Creek. Columns are organized by metrics, and rows are organized by parameters.

Table S12. Morris indices and 95% confidence intervals for the coefficient of variation and the timing and magnitude of peak streamflow for Spring Park Creek. Columns are organized by metrics, and rows are organized by parameters.

Table S13. Morris indices and 95% confidence intervals for the coefficient of variation and the timing and magnitude of peak streamflow for Upper Tenderfoot Creek. Columns are organized by metrics, and rows are organized by parameters.

Table S14. Morris indices and 95% confidence intervals for the coefficient of variation and the timing and magnitude of peak streamflow for Sun Creek. Columns are organized by metrics, and rows are organized by parameters.

Table S15. Morris indices and 95% confidence intervals for the coefficient of variation and the timing and magnitude of peak streamflow for Bubbling Creek. Columns are organized by metrics, and rows are organized by parameters.

Figure S3. Morris indices for the coefficient of variation and the timing and magnitude of peak streamflow, with σ (standard deviation index) plotted versus μ (mean index) across the five sub-catchments. Numbers refer to parameters as noted in Tables above. Sub-catchment abbreviations are Stringer Creek (STR), Spring Park Creek (SPC), Upper Tenderfoot Creek (UTC), Sun Creek (SUN), and Bubbling Creek (BUB).

Text S3. Morris results for soil moisture metrics.

Morris results are tabulated for average soil moisture across three soil layers (top/shallow, middle, bottom/deep) for Stringer Creek (Table S16), Spring Park Creek (Table S17), Upper Tenderfoot Creek (Table S18), Sun Creek (Table S19), and Bubbling Creek (Table S20). Indices are plotted against each other for each of these metrics and each sub-catchment in Figure S4.

Morris results are tabulated for the maximum soil moisture across three soil layers (top/shallow, middle, bottom/deep) for Stringer Creek (Table S21), Spring Park Creek (Table S22), Upper Tenderfoot Creek (Table S23), Sun Creek (Table S24), and Bubbling Creek (Table S15). Indices are plotted against each other for each of these metrics and each sub-catchment in Figure S5.

Morris results are tabulated for the minimum soil moisture across three soil layers (top/shallow, middle, bottom/deep) for Stringer Creek (Table S26), Spring Park Creek (Table S27), Upper Tenderfoot Creek (Table S28), Sun Creek (Table S29), and Bubbling Creek (Table S30). Indices are plotted against each other for each of these metrics and each sub-catchment in Figure S6.

Table S16. Morris indices and 95% confidence intervals for average soil moisture in the top/shallow, middle, and bottom/deep soil layers for Stringer Creek. Columns are organized by metrics, and rows are organized by parameters.

Table S17. Morris indices and 95% confidence intervals for average soil moisture in the top/shallow, middle, and bottom/deep soil layers for Spring Park Creek. Columns are organized by metrics, and rows are organized by parameters.

Table S18. Morris indices and 95% confidence intervals for average soil moisture in the top/shallow, middle, and bottom/deep soil layers for Upper Tenderfoot Creek. Columns are organized by metrics, and rows are organized by parameters.

Table S19. Morris indices and 95% confidence intervals average soil moisture in the top/shallow, middle, and bottom/deep soil layers for Sun Creek. Columns are organized by metrics, and rows are organized by parameters.

Table S20. Morris indices and 95% confidence intervals for average soil moisture in the top/shallow, middle, and bottom/deep soil layers for Bubbling Creek. Columns are organized by metrics, and rows are organized by parameters.

Figure S4. Morris indices for average soil moisture with σ (standard deviation index) plotted versus μ (mean index) top/shallow, middle, and bottom/deep soil layers for the five sub-catchments. Numbers refer to parameters as noted in Tables above. Sub-catchment abbreviations are Stringer Creek (STR), Spring Park Creek (SPC), Upper Tenderfoot Creek (UTC), Sun Creek (SUN), and Bubbling Creek (BUB).

Table S21. Morris indices and 95% confidence intervals for maximum soil moisture in the top/shallow, middle, and bottom/deep soil layers for Stringer Creek. Columns are organized by metrics, and rows are organized by parameters.

Table S22. Morris indices and 95% confidence intervals for maximum soil moisture in the top/shallow, middle, and bottom/deep soil layers for Spring Park Creek. Columns are organized by metrics, and rows are organized by parameters.

Table S23. Morris indices and 95% confidence intervals for maximum soil moisture in the top/shallow, middle, and bottom/deep soil layers for Upper Tenderfoot Creek. Columns are organized by metrics, and rows are organized by parameters.

Table S24. Morris indices and 95% confidence intervals for maximum soil moisture in the top/shallow, middle, and bottom/deep soil layers for Sun Creek. Columns are organized by metrics, and rows are organized by parameters.

Table S25. Morris indices and 95% confidence intervals for maximum soil moisture in the top/shallow, middle, and bottom/deep soil layers for Bubbling Creek. Columns are organized by metrics, and rows are organized by parameters.

Figure S5. Morris indices for maximum soil moisture with σ (standard deviation index) plotted versus μ (mean index) top/shallow, middle, and bottom/deep soil layers for the five sub-catchments. Numbers refer to parameters as noted in Tables above. Sub-catchment abbreviations are Stringer Creek (STR), Spring Park Creek (SPC), Upper Tenderfoot Creek (UTC), Sun Creek (SUN), and Bubbling Creek (BUB).

Table S26. Morris indices and 95% confidence intervals for minimum soil moisture in the top/shallow, middle, and bottom/deep soil layers for Stringer Creek. Columns are organized by metrics, and rows are organized by parameters.

Table S27. Morris indices and 95% confidence intervals for minimum soil moisture in the top/shallow, middle, and bottom/deep soil layers for Spring Park Creek. Columns are organized by metrics, and rows are organized by parameters.

Table S28. Morris indices and 95% confidence intervals for minimum soil moisture in the top/shallow, middle, and bottom/deep soil layers for Upper Tenderfoot Creek. Columns are organized by metrics, and rows are organized by parameters.

Table S29. Morris indices and 95% confidence intervals for minimum soil moisture in the top/shallow, middle, and bottom/deep soil layers for Sun Creek. Columns are organized by metrics, and rows are organized by parameters.

Table S30. Morris indices and 95% confidence intervals for minimum soil moisture in the top/shallow, middle, and bottom/deep soil layers for Bubbling Creek. Columns are organized by metrics, and rows are organized by parameters.

Figure S6. Morris indices for minimum soil moisture with σ (standard deviation index) versus μ (mean index) top/shallow, middle, and bottom/deep soil layers for the five sub-catchments. Numbers refer to parameters as noted in Tables above. Sub-catchment abbreviations are Stringer Creek (STR), Spring Park Creek (SPC), Upper Tenderfoot Creek (UTC), Sun Creek (SUN), and Bubbling Creek (BUB).

Text S4. Morris results for snow water equivalent metrics.

Morris results are tabulated for snow water equivalent metrics for Stringer Creek (Table S31), Spring Park Creek (Table S32), Upper Tenderfoot Creek (Table S33), Sun Creek (Table S34), and Bubbling Creek (Table S35). Indices are plotted against each other for each of these metrics and each sub-catchment in Figure S7.

Table S31. Morris indices and 95% confidence intervals for snow water equivalent metrics for Stringer Creek. Columns are organized by metrics, and rows are organized by parameters.

Table S32. Morris indices and 95% confidence intervals for snow water equivalent metrics for Spring Park Creek. Columns are organized by metrics, and rows are organized by parameters.

Table S33. Morris indices and 95% confidence intervals for snow water equivalent metrics for Upper Tenderfoot Creek. Columns are organized by metrics, and rows are organized by parameters.

Table S34. Morris indices and 95% confidence intervals for snow water equivalent metrics for Sun Creek. Columns are organized by metrics, and rows are organized by parameters.

Table S35. Morris indices and 95% confidence intervals for snow water equivalent metrics for Bubbling Creek. Columns are organized by metrics, and rows are organized by parameters.

Figure S7. Morris indices for soil moisture metrics with σ (standard deviation index) versus μ (mean index) top/shallow, middle, and bottom/deep soil layers for the five sub-catchments. Numbers refer to parameters as noted in Tables above. Sub-catchment abbreviations are Stringer Creek (STR), Spring Park Creek (SPC), Upper Tenderfoot Creek (UTC), Sun Creek (SUN), and Bubbling Creek (BUB).

Text S5.

We also include figures for results computed when forcing the model with uniform solar radiation. In these supplemental results, uniform solar radiation was varied according to a modifier that varied between 0.6 and 1, which was applied to the timeseries of solar radiation. This uniform modifier was used to account for basin variability in solar radiation due to topographic shading as well as the translation of a single point measurement across the catchments. Results displayed here are similar to results shown in the main text, which display sensitivity indices for forcing with spatially variable solar radiation. The largest differences can be observed for Upper Tenderfoot Creek.

Figure S8. Controls on (a) total annual or (b) growing season evapotranspiration. Abbreviations for sub-catchments are Stringer Creek (STR), Spring Park Creek (SPC), Upper Tenderfoot Creek (UTC), Sun Creek (SUN), and Bubbling Creek (BUB). The colors of the abbreviations (black/grey) correspond to the results for each sub-catchment.

Figure S9. Controls on (a) runoff ratio, (b) slope of the flow duration curve, (c) baseflow index and (d) coefficient of variation for streamflow across the sub-catchments. Abbreviations for sub-catchments are Stringer Creek (STR), Spring Park Creek (SPC), Upper Tenderfoot Creek (UTC), Sun Creek (SUN), and Bubbling Creek (BUB). The colors of the abbreviations correspond to the results for each sub-catchment.

Figure S10. Controls on (a) magnitude of peak runoff and (b) timing of peak runoff for streamflow across the sub-catchments. Abbreviations for sub-catchments are Stringer Creek (STR), Spring Park Creek (SPC), Upper Tenderfoot Creek (UTC), Sun Creek (SUN), and Bubbling Creek (BUB). The colors of the abbreviations correspond to the results for each sub-catchment.

Figure S11. Controls on average, minimum, and maximum soil moisture for the upper most (top), middle, and deepest (bottom) soil layers. Abbreviations for sub-catchments are Stringer Creek (STR), Spring Park Creek (SPC), Upper Tenderfoot Creek (UTC), Sun Creek (SUN), and Bubbling Creek (BUB). The colors of the abbreviations correspond to the results for each sub-catchment.

Figure S12. Controls on (a) peak snow water equivalent, (b) timing of maximum snow water equivalent, (c) snow water equivalent storage, and (d) maximum three-day melt. Abbreviations for sub-catchments are Stringer Creek (STR), Spring Park Creek (SPC), Upper Tenderfoot Creek (UTC), Sun Creek (SUN), and Bubbling Creek (BUB). The colors of the abbreviations correspond to the results for each sub-catchment.

Figure S13. Summary of primary, secondary, and tertiary importance sensitivity indices for Stringer Creek. Circles indicate the degree of interactions.

Figure S14. Summary of the parameter types (snow, canopy, undergrowth, soil) that are primary, secondary, or tertiary controls on model output metrics.
